# Prediction of peptidoglycan hydrolases- a new class of antibacterial proteins

**DOI:** 10.1186/s12864-016-2753-8

**Published:** 2016-05-27

**Authors:** Ashok K. Sharma, Sanjiv Kumar, Harish K., Darshan B. Dhakan, Vineet K. Sharma

**Affiliations:** Metagenomics and Systems Biology Group, Department of Biological Sciences, Indian Institute of Science Education and Research Bhopal, Bhopal, 462066 India; Department of Medicine, University of Connecticut Health Center, Farmington, CT 06030 USA

**Keywords:** Peptidoglycan hydrolase, N-acetylglucosaminidase, N-acetylmuramidases, Lytic transglycosylases, Endopeptidase, N-acetylmuramoyl-L-alanine, Carboxypeptidase, Cell wall hydrolases, Support Vector Machine, Random Forest

## Abstract

**Background:**

The efficacy of antibiotics against bacterial infections is decreasing due to the development of resistance in bacteria, and thus, there is a need to search for potential alternatives to antibiotics. In this scenario, peptidoglycan hydrolases can be used as alternate antibacterial agents due to their unique property of cleaving peptidoglycan cell wall present in both gram-positive and gram-negative bacteria. Along with a role in maintaining overall peptidoglycan turnover in a cell and in daughter cell separation, peptidoglycan hydrolases also play crucial role in bacterial pathophysiology requiring development of a computational tool for the identification and classification of novel peptidoglycan hydrolases from genomic and metagenomic data.

**Results:**

In this study, the known peptidoglycan hydrolases were divided into multiple classes based on their site of action and were used for the development of a computational tool ‘HyPe’ for identification and classification of novel peptidoglycan hydrolases from genomic and metagenomic data. Various classification models were developed using amino acid and dipeptide composition features by training and optimization of Random Forest and Support Vector Machines. Random Forest multiclass model was selected for the development of HyPe tool as it showed up to 71.12 % sensitivity, 99.98 % specificity, 99.55 % accuracy and 0.80 MCC in four different classes of peptidoglycan hydrolases. The tool was validated on 24 independent genomic datasets and showed up to 100 % sensitivity and 0.94 MCC. The ability of HyPe to identify novel peptidoglycan hydrolases was also demonstrated on 24 metagenomic datasets.

**Conclusions:**

The present tool helps in the identification and classification of novel peptidoglycan hydrolases from complete genomic or metagenomic ORFs. To our knowledge, this is the only tool available for the prediction of peptidoglycan hydrolases from genomic and metagenomic data.

Availability: http://metagenomics.iiserb.ac.in/hype/ and http://metabiosys.iiserb.ac.in/hype/.

**Electronic supplementary material:**

The online version of this article (doi:10.1186/s12864-016-2753-8) contains supplementary material, which is available to authorized users.

## Background

The compounds which act against bacterial infection either by suppressing its growth or by killing the bacterium are mainly considered as antibacterial agents such as sulfonamide derivatives and tetracycline antibiotic [1]. These antibiotics have been widely used as medicines for humans and animals for fighting against bacterial infection. However, in the last decades, these antibiotics have not shown consistent effectiveness against bacterial infections due the emergence of drug resistance in bacteria against these antibiotics [2]. This problem poses a serious challenge towards the researchers to discover either newer drug molecules with lower bacterial drug resistance or to look for the alternatives of antibiotics [3]. Recently, peptidoglycan hydrolases have been proposed as potential alternative for antibiotics due to their bacteriolytic activity with multifarious spectrum [4–6]. Among the various sites of action of the antibacterial agents, bacterial cell wall has been a widely used target which is also the target site for peptidoglycan hydrolases [7].

The bacterial cell wall is made up of glycan strands which are cross-linked by flexible peptide side chains, providing strength and rigidity to the bacterial cell wall [8]. The peptidoglycan of both gram-positive and gram-negative bacteria comprises of repeating units of N-acetylglucosamine (NAG) and β-(1–4)-N-acetylmuramic acid (NAM) cross-linked by peptide stem chains attached to the NAM residues [9]. First two peptides of tetra-peptide chain generally consist of L-alanine and D-glutamine or isoglutamine and the last residue is generally D-alanine. The third residue of stem peptide varies across bacteria and is lysine in coccoid gram-positive bacteria and meso-diaminopimelate (mDAP) in gram-negative bacteria and in many gram-positive rods such as *Listeria* and *Bacillus* species [10]. The peptidoglycan layer is highly dynamic during cell growth and reshapes on division.

**Fig. 1:**
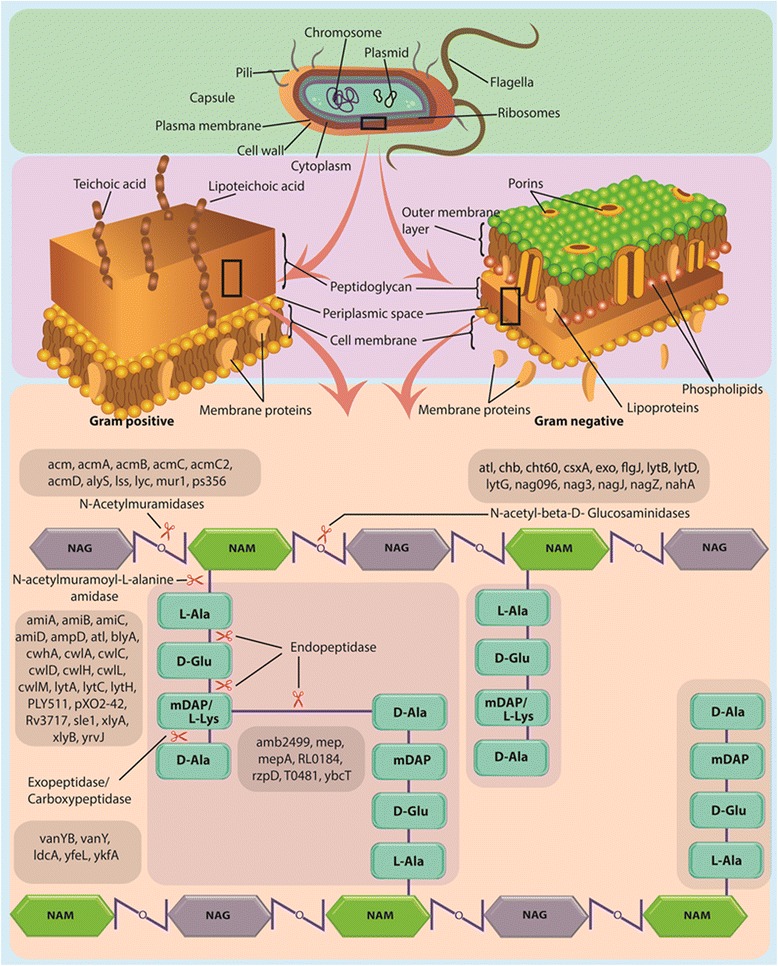
Sites of action of peptidoglycan hydrolases on bacterial cell wall

Bacterial peptidoglycan hydrolases are the enzymes responsible for cleaving the bonds in peptidoglycan chain and side-chain branches, therefore, are responsible for maintaining overall cell wall peptidoglycan turnover [11, 12]. Three main classes of bacterial peptidoglycan hydrolases are glycosidases that cleave the backbone of glycan, the amidases that cleave the side-chain peptide and peptidases (endopeptidases and carboxypeptidases) that cleave within the peptide side-chain, which are further divided based on their site of cleavage [13, 14]. The glycosidases consists of N-acetylglucosamidases which hydrolyses N-acetyl-D-glucosamine (GlcNAc) residues from contiguous sugar residues and N-acetylmuramidases cleaves the β1-4 glycosidic bond between N-Acetylmuramic acid (MurNAc) and GlcNAc. There are two enzymatic methods which can carry out the cleavage of bond between MurNAc and GlcNAc, i.e., lysozyme glycosidic cleavage which results in generation of terminal MurNAc residue, and lytic transglycosylases which forms 1, 6-anhydro ring on MurNAc residue [13, 15]. On the other hand amidases consists of N-acetylmuramyl-L-alanine amidases, cleaving the bond between peptide side chain and glycan strand, endopeptidases, cleaving amide bond in between two amino acid residues in a peptide chain, and carboxypeptidases, cleaving the bond at peptide terminal in a peptide chain [13]. The endopeptidases and carboxypeptidases are referred to as DD-peptidases if they cleave the bond between D-amino acid, and are referred to as DL or LD-peptidases if they cleave the bond between D- and L- amino acids [13]. Schematic representation of peptidoglycan hydrolases is shown in Fig. 1.

Several studies have been carried out on cell wall autolysins (peptidoglycan hydrolases) in various bacterial populations with roles pertaining to the peptidoglycan turnover along with the other functions in bacteria. Though potentially lethal, these autolysins are universally present among bacteria that have peptidoglycan. Lysostaphin is one of the most studied peptidoglycan hydrolase, excreted by *Staphylococcus simulans* cleaving the peptidoglycan chain of *Staphylococcus aureus* without affecting itself [16]. Zoocin A which is produced by *Streptococcus zooepidemicus* 4881 is also a bacteriolytic cell wall hydrolase similar to lysostaphin [17]. It has recently been demonstrated to be potentially effective in controlling and treating infection caused by *Staphylococcus aureus* group of bacteria. Millericin B which is another antimicrobial murein hydrolase produced by *Streptococcus milleri* NMSCC061 inhibits the growth of several bacterial species [18]. A muraminidase Cpl-1 is also a phase lytic enzyme and was used for the first time to treat pneumococcal meningitis infection through intravenous administration [19]. Antimicrobial properties of peptidoglycan hydrolase (such as lysozyme) have been known since several decades [20]. The efficacy of lysozyme has been shown for skin treatment and in the infections of mucus membranes and is used as an ingredient in wound healing ointments [21, 22]. Bacteriophage endolysin PlyC in an aerosolized form was active against pathogenic *Streptococcus equi* and is considered as the first narrow-spectrum disinfectant against the bacterial strain [23]. Endolysin PlyV12 and Enterolysin A are known peptidoglycan hydrolase having anti-enterococcal activity [24, 25]. Several other peptidoglycan hydrolases such as Acd, LytA, or PL-1 were identified and purified from various origins having antibacterial activity mainly against gram-positive bacteria [26–28]. Taken together, these studies underscore the potential of using peptidoglycan hydrolases as antibacterial agents in several applications including therapeutics.

Identification and classification of novel peptidoglycan hydrolases in the completely sequenced genomes becomes difficult due to the lack of homology of these hydrolases with the previously well characterized peptidoglycan hydrolases. Therefore, in the present work, a machine learning based approach using Random Forest (RF) has been used and demonstrated for the identification and classification of novel peptidoglycan hydrolases from genomic and metagenomic data. The predicted novel peptidoglycan hydrolases belonging to different classes would provide leads for further characterization and potential application as antibacterial agents specifically against various bacterial species.

## Results and discussion

### Selection of machine learning method

To select appropriate machine learning method and feature inputs for construction of final module providing the most accurate classification, the Amino Acid Composition (AAC) and Dipeptide Composition (DPC) were used as features calculated from randomly selected 10 % data from the positive and negative datasets. At the first level of binary classification, the idea was to optimize the parameters for construction of a module which can predict peptidoglycan hydrolases from input sequences. The selected (10 %) data was used to carry out ten-fold cross-validation in WEKA. It is apparent from Additional file 1 that LibSVM performed better than other machine learning techniques as it showed the highest accuracy of 93.15 % in the case of DPC features and 91.3 % in case of AAC features. RF also showed good results in case of both AAC (86.64 %) and DPC features (86.62 %).

At the second level, termed as ‘multiclass classification’, for a protein predicted as peptidoglycan hydrolase, the category of the protein is predicted using the information from the five categories (see Methods) based on their site of action. AAC features and DPC features of randomly selected 10 % data from the total dataset, where each sequence was tagged with its respective category, were used for evaluation using ten-fold cross-validation using WEKA. LibSVM performed better than the other machine learning methods and showed the highest accuracy of 90.01 % for DPC features and 87.68 % for AAC features. RF also showed good results with an accuracy of 83.45 % in case of DPC as feature input, and 82.63 % when AAC was used as feature input (Additional file 2).

These results suggest that both LibSVM and RF, using AAC and DPC features and on using 10 % of the total data, performed better than the other machine learning methods, and can be further evaluated and optimized on the complete dataset. The further optimization was carried out to select the best features and a machine learning method for construction of the final module.

### Optimization of LibSVM and random forest modules

#### For binary classification

**Fig. 2:**
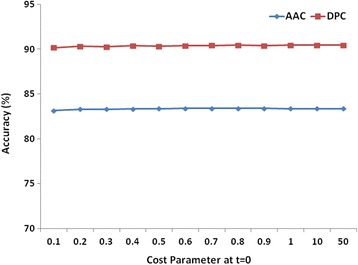
Performance of LibSVM modules using linear kernel at different cost parameters for AAC and DPC for binary classification

The performance of LibSVM was optimized using both AAC and DPC as the feature input. After fine tuning of various parameters, it was observed that linear kernel with DPC feature based modules, at all possible c values, performed better than AAC feature based modules (Fig. 2). The five-fold cross validation was carried out for performance evaluation at c = 1. At threshold of zero (default), sensitivity (78.76 %), specificity (94.67 %), accuracy (90.4 %) and MCC (0.75) obtained using DPC features based module was higher compared to the sensitivity (53.81 %), specificity (93.63 %), accuracy (82.94 %) and MCC (0.53) obtained using AAC features based modules (Table 1). Therefore, DPC feature input with the linear kernel at a cost parameter of 1 was considered for construction of the final LibSVM module. This LibSVM module was further compared with the optimized modules of RF for binary classification at the first level to choose more accurate classifier between these two methods.

**Table 1:** Comparative performance of LibSVM and RF using Amino Acid and Dipeptide as feature inputs for binary classification

	Sensitivity		Specificity		Accuracy		MCC	
	AAC	DPC	AAC	DPC	AAC	DPC	AAC	DPC
SVM	53.81	78.76	93.63	94.67	82.94	90.40	0.53	0.75
RF	74.14	75.08	97.79	99.48	91.44	92.93	0.77	0.82

AAC = Amino acid composition and DPC = Dipeptide composition

SVM = Support Vector Machine at t = 0 and c = 1 for both AAC and DPC

RF = Random Forest; for AAC mtry = 4, ntree = 500 and for DPC mtry = 64, ntree = 500

**Fig. 3:**
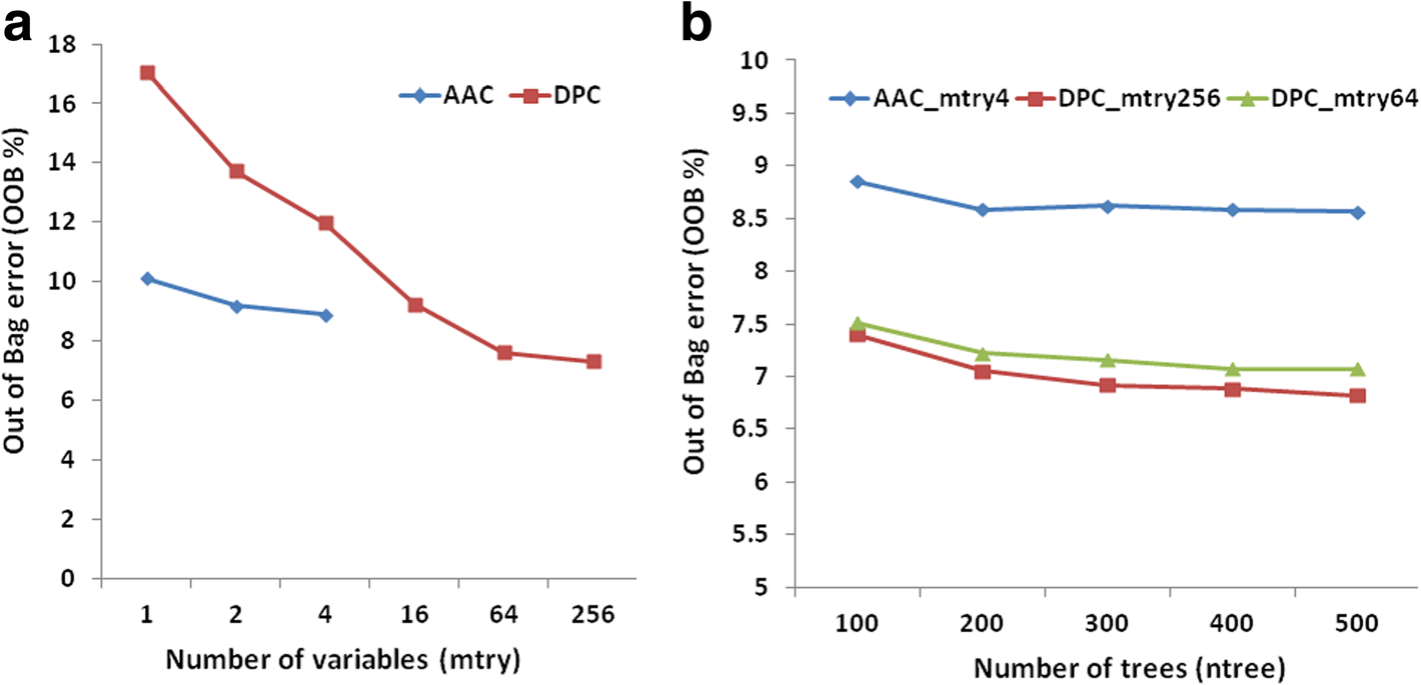
**a** OOB error using AAC and DPC as feature inputs at different mtry for binary classification. **b** OOB error on increasing the number of trees at optimized mtry for AAC and DPC for binary classification

The performance of RF was optimized separately in R package using both AAC and DPC features as an input vector. Optimization of mtry (random variables at each split node) was carried out using tuneRF function of the RF at ntree = 100. OOB error was minimum (8.9 %) at mtry = 4 using AAC as the feature input, and OOB error was minimum at mtry = 64 (7.63 %) and at mtry = 256 (7.31 %) using DPC as the feature input (Fig. 3a). Further optimization was carried out using more number of trees at optimized mtry values for both AAC and DPC. The final module constructed using AAC as a feature at mtry = 4 and ntree = 500 displayed the OOB error of 8.56 %, and the module constructed using DPC as a feature at mtry = 64 and mtry = 256 using ntree = 500 displayed the OOB error of 7.07 and 6.82 %, respectively (Fig. 3b). It is apparent that OOB error was lower for DPC as compared to AAC, therefore DPC was selected as the feature input. Since the difference in OOB error at mtry = 64 and mtry = 256 using DPC was very low (0.25 %), the mtry = 64 was selected for the final module construction. The performance of the final modules of RF and LibSVM was comparable, however, the LibSVM module showed higher sensitivity (78.76 %) and the RF module showed higher specificity (99.48 %), accuracy (92.93 %) and MCC (0.82 %) (Table 1). Therefore, both the modules were considered for binary classification and were further evaluated at the time of construction of final pipeline of the prediction tool for Hydrolases of Peptidoglycans ‘Hype’.

#### For multiclass classification

**Fig. 4:**
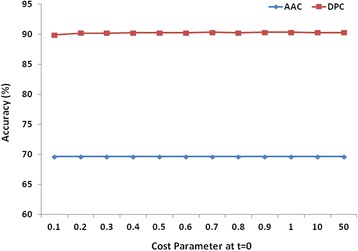
Performance of LibSVM modules using linear kernel at different cost parameters for AAC and DPC for multiclass classification

For the development of multiclass classifier, the performance of LibSVM was optimized using the same feature inputs used in the first level for binary classification. LibSVM module using linear kernel performed better for DPC in comparison to AAC features at all possible c values (Fig. 4). Using five-fold cross validation using c = 0.7, at zero threshold, the sensitivity, accuracy and MCC values for the peptidoglycan hydrolases class predictions was lower for AAC features based module as compared to DPC based modules (Table 2). Therefore, DPC with the linear kernel was considered for LibSVM and was further compared with the results of RF.

**Table 2:** Performance of LibSVM using Amino acid and Dipeptide composition as feature inputs for multiclass classification

Class	Sensitivity		Specificity		Accuracy		MCC	
	AAC	DPC	AAC	DPC	AAC	DPC	AAC	DPC
A	7.53	76.57	97.95	97.16	86.67	94.67	0.07	0.74
B	0	52.45	100	99.6	96.91	98.31	0	0.62
C	14.2	55.88	98.36	97.55	88.94	93.47	0.19	0.6
D	0	32.77	100	99.81	99.37	99.4	0	0.4
E	98.23	96.17	15.49	75.08	76.01	90.74	0.26	0.74

SVM = Support Vector Machine at t = 0 and c = 0.7 for both AAC and DPC

Where, A = N-acetylmuramoyl-L-alanine amidases, B = Peptidases, C = Enzymes acting on Peptidoglycan chain, D = Unclassified, and E = Negative Dataset

**Fig. 5:**
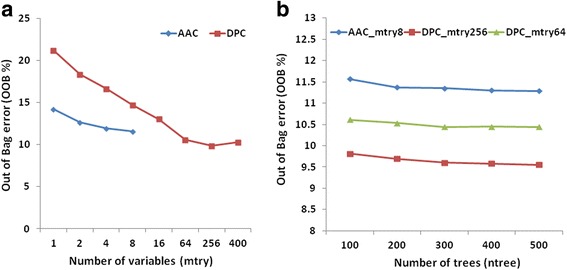
**a** OOB error using AAC and DPC as feature inputs at different mtry for multiclass classification. **b** OOB error on increasing the number of trees at optimized mtry for AAC and DPC for multiclass classification

The performance of RF was optimized at different mtry values using both AAC and DPC features as input using the tuneRF function. Using AAC, the OOB error was minimum (11.55 %) at mtry = 8, on using DPC the OOB error was minimum at mtry = 64 (10.57 %) and mtry = 256 (9.85 %) (Fig. 5a). Further optimization was carried out by increasing the number of trees (ntree = 100 to 500) at optimized mtry for both AAC and DPC features.

The module constructed using AAC as the feature input at mtry = 8 and ntree = 500 displayed an OOB error of 11.29 %, and the module constructed using DPC as the feature input at mtry = 64 and mtry = 256 using ntree = 500 displayed OOB errors of 10.44 and 9.55 %, respectively (Fig. 5b). A significant difference was not observed between the OOB errors obtained after the increment in mtry values from 64 to 256. The performance of AAC and DPC modules is shown in Table 3. Therefore, DPC at mtry = 64 was considered for the construction of DPC based module.

**Table 3:** Performance of Random Forest (RF) final models using Amino acid and Dipeptide composition as feature inputs for multiclass classification

Class	Sensitivity		Specificity		Accuracy		MCC	
	AAC	DPC	AAC	DPC	AAC	DPC	AAC	DPC
A	62.52	71.12	98.55	99.47	93.90	95.77	0.70	0.80
B	61.10	54.91	99.95	99.98	98.62	98.45	0.77	0.73
C	59.73	53.49	98.72	99.66	94.19	94.34	0.68	0.69
D	40.41	38.19	99.97	99.98	99.55	99.55	0.60	0.60
E	98.99	99.93	64.50	62.92	90.14	90.18	0.73	0.74

Where, A = N-acetylmuramoyl-L-alanine amidases, B = Peptidases, C = Enzymes acting on Peptidoglycan chain, D = Unclassified, and E = Negative Dataset

From the optimization results, it is evident that at the first level of classification the performances of both LibSVM and RF modules were comparable; however, at the second level the RF module performed better than LibSVM module (Tables 1, 2 and 3). Therefore, for the multiclass classification RF module was considered as the final classifier, and for the binary classification further evaluation was carried out to select between the LibSVM and RF modules. Using these modules, three prediction approaches were constructed using the following strategy.

In the first approach, at the first level (for binary classification) the LibSVM module was implemented using DPC as the feature input and linear kernel at cost factor of 1. The query proteins were classified as ‘positive’ or ‘negative’ hits at this level and all the positive hits were further used as query protein at the second level (for multiclass classification). At the second level, the RF module constructed using DPC at mtry = 64 and ntree = 500 was used for the classification of positive hits (predicted peptidoglycan hydrolases) obtained from the first level.

The second approach was also implemented using the same methodology, however, at the first level the RF module constructed using DPC at mtry = 64, ntree = 500 has been used for binary classification in place of LibSVM module. The RF module at second level remained the same as mentioned in the first approach. The query proteins were analyzed using the same procedure as mentioned in the first tool.

The third approach had only a single level where the RF module constructed using DPC at mtry = 64 and ntree = 500 was used for the classification of query proteins into the five categories as mentioned in Additional file 3.

### Performance evaluation of the three approaches

Two datasets were used to examine the performance of the three approaches. The first dataset was constructed using randomly selected 250 known peptidoglycan hydrolases from 24 bacterial genomes (from validation set) and was used as a query to evaluate the performance of the three approaches. The performance was evaluated in terms of the number of peptidoglycan hydrolases which could be predicted positively and the time required for the prediction by each tool. It is apparent that the third approach which used only the RF module for multiclass classification performed better (220 correct predictions out of 250 proteins in 2.90 s) as compared to the other two approaches (Additional file 4).

The complete ORFs predicted from the metagenomic dataset (MH0016) were used as the second dataset for the performance evaluation. BLAST was performed for these ORFs against the positive dataset and the ORFs which showed significant (E <1e-6 and identity ≥80 %) best hits with the positive dataset were considered as true peptidoglycan hydrolases. These selected 41 ORFs were further used to evaluate the results obtained from the three approaches. In this case also, the performance of the third approach was better (30 correct predictions out of 41 in 19 s) than the other two approaches (Additional file 5). It is evident from these results that the third approach which involved only one level of classification using RF module showed the best performance among all the three approaches and, hence was selected for developing the prediction tool termed as ‘HyPe’.

### Validation of ‘HyPe’ on independent genomic datasets

The first independent dataset consisted of protein sequences from 24 new bacterial genomes and was used to evaluate the performance of HyPe. The annotated peptidoglycan hydrolases from each genome was used as the reference dataset to compare the predictions made by HyPe and BLAST. To evaluate the performance of BLAST, local alignment was carried out for the proteins present in each genome against the positive dataset. The proteins which showed significant (E <1e^−6^ and identity ≥80 %) similarity with the positive dataset were selected as positive hits. Similarly, the proteins from each of the 24 bacterial genomes were analyzed using HyPe to predict peptidoglycan hydrolases from each genome.

Out of all the 24 genomes, BLAST predicted the maximum (30) number of peptidoglycan hydrolases for the genome *Bacillus anthracis strain PAK1* and the ‘HyPe’ predictions were maximum (50) for *Cronobacter sakazakii SP291*. For the genome *Chlamydia trachomatis* strain L2b CS19_08, only a single peptidoglycan hydrolase was predicted using both BLAST and ‘HyPe’. Some of the peptidoglycan hydrolase proteins (as per their annotation in the genomes) could not be predicted by BLAST and ‘HyPe’. The maximum number of such proteins was 10 for genome *Bacillus subtilis* T30 (Additional file 6). Similarly, HyPe could predict several peptidoglycan hydrolases which could not be predicted by BLAST. Therefore, the performance of HyPe for all 24 bacterial genomes was evaluated by adding the number of peptidoglycan hydrolases which were commonly predicted by both BLAST and HyPe and the new correct predictions of HyPe which are together called as ‘true positive’. The incorrect predictions of HyPe were called as ‘false positive’ and the peptidoglycan hydrolases which could not be correctly predicted by HyPe were called as ‘false negative’. The remaining protein sequences in a given genome were called as ‘true negative’. Sequences of hypothetical and putative proteins were not considered at the time of performance evaluation. The detailed performance evaluation for the genomic dataset is provided in Table 4.

**Table 4:** Performance of HyPe on independent genomic dataset

Genome	Sensitivity	MCC
*Alcanivorax pacificus* RT type strain W11 5	71.43	0.56
*Bacillus anthracis* strain PAK 1	77.42	0.81
*Bacillus cere*us strain 03BB87	75.86	0.77
*Bacillus subtilis* T30	62.96	0.67
*Bacillus thuringien*sis strainHD571	84.00	0.86
*Campylobacter je*juni subsp jejuni strain YH001	42.86	0.51
*Chlamydia trachomatis* strainL2b CS19 08	50.00	0.71
*Clostridium botulinum* strain NCTC8550	68.18	0.75
*Cronobacter sakazakii* SP291	85.71	0.74
*Francisella tularens*is subsp. novicida U112	100.00	0.82
*Haemophilus influenzae strain Hi375*	82.35	0.88
*Halomonas sp* strain KO116	88.89	0.94
*Lactobacillus sp.* wkB8	66.67	0.63
*Listeria monocytogenes* Serovar 4b Strain IZSAM Lm hs2008	92.31	0.86
*Listeria monocytogenes* strain NTSN	80.00	0.77
*Mycobacterium tuberculosis* H37RvSiena	100.00	0.78
*Neisseria meningitidis* LNP21362	78.57	0.65
*Pasteurella multocida* OH1905	62.50	0.59
*Rickettsia rickettsii* str Morgan	75.00	0.75
*Staphylococcus aureus* strain FCFHV36	77.27	0.81
*Streptococcus iniae* strain ISNO	60.00	0.63
Vibrio alginolyticus NBRC 15630	64.71	0.74
*Vibrio tubiashii* ATCC 19109	70.59	0.75
*Weissella ceti strain WS74*	60.00	0.67

The Specificity and Accuracy was almost 1 for all the above genomes since the number of True Negatives (TN) was very large in number, which used in the denominator for the calculation to Specificity and Accuracy

### Validation of ‘HyPe’ on real metagenomic datasets

The second independent dataset consisted of 24 metagenomic samples obtained from the Human Gut Microbial Gene Catalogue and processed using the methodology discussed in the methods section (Additional file 7). The complete proteins having length ≥100 amino acids were used as query for each metagenome. Using BLAST, the maximum (83) number of peptidoglycan hydrolases were predicted for metagenomic sample MH0045, whereas, using HyPe the maximum (288) number of peptidoglycan hydrolases were predicted for metagenomic sample MH0085, among all metagenomic samples. The maximum number of common predictions by BLAST and HyPe was 79 for MH0074. The complete results from the comparison of BLAST and HyPe on all 24 metagenomic samples are provided in Additional file 8.

### Development of the HyPe pipeline

The web server for HyPe is developed using the standalone HyPe application which can be used for the identification of peptidoglycan hydrolases from complete genomic or metagenomic ORFs (Additional file 9). Query sequence will pass through the RF module to predict positive hits (peptidoglycan hydrolases) and also to categorize the resultant peptidoglycan hydrolases into their respective classes.

For the analysis of genomic and metagenomic proteins, separate options ‘Genomic’ and ‘Metagenomic’ has been provided at ‘Application’ page. The user should upload the protein sequence file in FASTA format using the ‘Genomic’ option. The ORFs (in FASTA format) predicted by any gene prediction software should be uploaded using the ‘Metagenomic’ option. The page with the ‘Job ID’ will be displayed to access the results after submission of a query. The standalone version of HyPe is developed for usage on the Linux OS based computer and requires the installation of free packages such as R and Random Forest (Details on Download page on website http://metagenomics.iiserb.ac.in/hype, http://metabiosys.iiserb.ac.in/hype, and in Additional file 10.

### Prediction of peptidoglycan hydrolases from pathogenic bacteria using HyPe webserver

Forty-five pathogenic and 3 non-pathogenic (*Bdellovibrio bacteriovorus*, *Bifidobacterium animali* and *Deinococcus radiodurans*) genomes were analyzed using HyPe web server to identify the peptidoglycan hydrolases. The list of predicted peptidoglycan hydrolases from these most common pathogenic bacterial species is provided in Additional file 11. Some of the peptidoglycan hydrolases with well-established antibacterial activity could be identified using HyPe, such as Lysostaphin from *Staphylococcus simulans* with activity against *Staphylococcus aureus* [16], LasA from *Pseudomonas aeruginosa* [29] with lytic activity against various *Staphylococcus* species (i.e., *S. saprophyticus*, *S. epidermidis* and *S. warneri*) [30], PlyL from *Bacillus anthracis* which cleaves the cell wall of several *Bacillus* species when applied exogenously [31], N-acetylmuramoyl-L-alanine amidase from various organisms which has lytic effect with varying spectrum of activity [6, 32–34], and lytic activity of lysozymes which has been known since long time [35]. Large number of peptidoglycan hydrolases could be predicted in various gram-positive and gram-negative bacterial species, most of them being hypothetical proteins (205 of 1203) (Additional file 11), enabling identification and characterization of novel cell wall hydrolases. With the wide spectrum of activity of these proteins it would be possible to customize the usage of peptidoglycan hydrolases according to the type of antibacterial spectral requirement. It is also plausible to believe that exploration of this class of antibacterial proteins from metagenomic data would lead to identification of novel cell wall hydrolases with desired antibacterial spectra and activity.

## Conclusions

The novel strategies for antibacterial development are requisite to tackle the ongoing struggle between emergence of resistance and slow development of new antibiotics. Only a few peptidoglycan hydrolases have yet been identified from completely sequenced genomes as potential antibacterial agents. However, from different metagenomic datasets, more diversely active (with both narrow and broad range spectrum of activity as murein hydrolase) peptidoglycan hydrolases could be identified which is an unexplored area for this class of proteins. These novel peptidoglycan hydrolases have the potential to be used, as shown previously, in food industry for preservation, in agriculture for achieving resistance against phytopathogenic bacteria, and as antibacterial agents [36, 37]. The peptidoglycan hydrolases could be developed into a new class of antibacterial agents to counteract the problem of antibiotic resistance in pathogenic organisms [38–41]. Identification and characterization of novel peptidoglycan hydrolases will also provide insights into better understanding of pathophysiology of various pathogens. To the best of our knowledge, this is the only tool available to predict the peptidoglycan hydrolases from genomic and metagenomic data.

## Methods

### Construction of datasets

#### Construction of peptidoglycan hydrolase dataset and negative dataset

A total of 399,933 sequences were retrieved from NCBI protein database (website) using the following terms n-acetylmuramidases, n-acetylmuramoyl-l-alanine amidase, n-acetylglucosaminidase, and “murein and carboxypeptidase” “murein and endopeptidase” to obtain the peptidoglycan hydrolases from different bacterial origin. Sequences with ambiguous terms (hypothetical, like, similar, related, unknown, possible, probable, putative, partial, uncharacterized, predicted, inhibitor, regulator, enhancer, unnamed, precursor, fraction, and repressor) in annotations were removed and not considered for further analysis. A total of 281,313 sequences which remained after the removal of ambiguous terms were clustered at 95 % identity using CD-HIT [42].

The resulting dataset consisting of 62,572 representative sequences was labeled as the positive dataset. To construct the negative dataset, 547,085 protein sequences were retrieved from UniProtKB/Swiss-Prot database (http://www.uniprot.org/downloads, version 20 Nov 2014) [43]. The sequences with annotations containing the search terms used to construct the positive dataset were removed. Clustering was performed for the remaining sequences in the negative dataset at 80 % identity using CD-HIT to avoid over-representation of similar sequences. The resultant dataset consisted of 195,220 sequences. From this dataset, in order to remove sequences which could be remotely related to the positive dataset, BLAST was performed at e-value < 10 using the sequences of the positive dataset as query sequence [44]. All those sequences which showed a similarity with the sequences in the positive dataset were removed and the remaining sequences were considered as the negative dataset. For appropriate training, the sequences smaller than 100 amino acids were removed from the positive and negative datasets. The final positive and negative datasets contained 23,062 and 62,837 sequences, respectively.

Positive dataset were manually curated to classify the peptidoglycan hydrolases sequences into three main categories depending on the site of action at peptidoglycan. The three representative categories were N-acetylmuramoyl-L-alanine amidase (acting on bond between the tetra-peptide side chain and peptidoglycan chain) (NAMLAAmidases), murein peptidases (including endopeptidases and carboxypeptidases) (Peptidases) and the enzymes acting on peptidoglycan chain (N-acetylmuramidases, N-acetylglucosaminidase) (Peptidoglycan_Chain). Remaining sequences which could not be classified into any of the three categories were collectively considered as unclassified peptidoglycan hydrolases and constituted the fourth category (Additional file 3).

#### Independent genomic datasets

For the performance evaluation of the prediction method, an independent set was created using 24 new bacterial genomes which were released at EMBL-EBI (http://www.ebi.ac.uk/genomes/bacteria.html) during January to May 2015. The positive and negative datasets constructed in the earlier sections contained sequences from genomes which were released earlier to given time period. Thus, it eliminates any chances of biasness in the predictions by the tool since the sequences in the independent set were not included in the training set. Each genome from the independent set was analyzed using the prediction tool to identify peptidoglycan hydrolases. BLAST was performed for each genome against the positive dataset and the results of BLAST were compared with the results of the prediction tool. Further, the peptidoglycan hydrolases were manually identified from each genome using same the set of keywords (positive set) as used in the previous section to identify all known peptidoglycan hydrolases in that genome.

#### Metagenomic datasets

Twenty-four metagenomic samples were obtained from the Human Gut Microbial Gene Catalogue [45] (Additional file 7). The paired-end reads from each sample were assembled in to a single read of average length 131 bp by FLASH [46] and were further assembled into contigs using MEGAHIT [47]. ORF predictions were carried out in contigs using MetaGeneMark [48] and only complete ORFs (with start and stop codon) having length ≥100 amino acids were used for the evaluation of prediction tool.

### Feature extraction

#### Amino acid and dipeptide composition

Amino-acid composition (AAC) and Dipeptide composition (DPC) were used as features and were calculated using in-house Perl scripts in the positive and negative datasets. Sequence belonging to the positive and negative datasets were labeled as “+1” and “-1” respectively. The AAC and DPC features of each protein were calculated using the following formula [49].

$$ AAC(i)=\frac{\mathrm{Total}\ \mathrm{number}\ \mathrm{of}\ \mathrm{amino}\ \mathrm{acid}\ \left(\mathrm{i}\right)}{\mathrm{Total}\ \mathrm{number}\ \mathrm{of}\ \mathrm{all}\ \mathrm{possible}\ \mathrm{amino}\ \mathrm{acid}\mathrm{s}} \times 100 $$

where, amino acid (i) is one of the 20 amino acids and AAC(i) is the amino acid composition of the amino acid (i).

$$ DPC(i)=\frac{\mathrm{Total}\ \mathrm{number}\ \mathrm{of}\ \mathrm{dipeptides}\ \left(\mathrm{i}\right)}{Total\ \mathrm{number}\ \mathrm{of}\ \mathrm{all}\ \mathrm{possible}\ \mathrm{dipeptides}} \times 100 $$

where, the dipeptide (i) is one out of 400 dipeptides and DPC(i) is the dipeptide frequency of dipeptide (i).

### Machine learning techniques

#### Selection of machine learning method

In the preliminary analysis, functional domains were also searched in the peptidoglycan hydrolase protein sequences in order to apply HMM, however, domain based approach could not be implemented due to the reasons mentioned in Additional file 12.

The AAC and DPC as features and the different machine learning approaches were evaluated using WEKA, which provides several machine learning algorithms for classification, regression and clustering analysis [50]. From the positive and negative dataset, only 10 % of the data was used for the evaluation at first level termed as binary classification. The first level is for the binary classification of a protein as peptidoglycan hydrolase or other function. After the first level where a protein is identified as peptidoglycan hydrolase, the second level is for the classification of this protein into the various categories based on the site of action of peptidoglycan hydrolases. For multi-class classification, the sequences in positive dataset which were classified into four categories were labeled as A, B, C and D, and the negative dataset were labeled as E (Additional file 3) and only 10 % of the data was used for the evaluation at the second level.

#### Support Vector Machine (SVM)

SVM was implemented via LibSVM package (http://www.csie.ntu.edu.tw/~cjlin/libsvm/) [51]. It is a supervised machine learning algorithm which is used to distinguish the data based on observed patterns within the data. LibSVM provides the flexibility to optimize the number of parameters and kernels [52, 53]. The kernel which provided better results at the time of optimization was considered for the construction of SVM module for the development of prediction tool. To evaluate the unbiased performance of the LibSVM, five-fold cross-validation experiment was performed.

#### Random Forest (RF)

RF was implemented using the randomForest package provided in R (http://cran.r-project.org//) [54]. RF is one of the implementation of an ensemble-learning method based on the construction of decision trees for classification and regression [55]. Each tree in the forest works as independent model and the output depends upon the overall performance. This algorithm shows better performance as compared to other machine learning methods since several model works together in this approach and the final class is predicted via overall decisions given by individual models. The mode of classification is decided by bootstrapping of classification trees, by choosing a mtry value (which decides the number of variables to be used at each node to split) and trying to minimize the out of bag (OOB) error rate. The OOB error mainly depends upon the strength of the relationship between the trees and strength of each tree [54, 56]. The performance of RF was assessed using various parameters such as mtry and ntree. The parameters which showed the best performance were used for the final RF module construction for the development of prediction tool.

#### Comparison with BLAST

BLAST (version 2.2.26) was used in this study to compare the performance of the prediction tool on genomic and real metagenomic datasets.

### Cross-validation and performance evaluation

The performance of LibSVM and RF was evaluated using the parameters discussed below.

$$ Accuracy=\frac{TP+TN}{TP+FN+FP+TN} $$

$$ Sensitivity=\frac{TP}{TP+FN} $$

$$ Specificity = \frac{TN}{TN+FP} $$

$$ MCC = \frac{\left(TP \times TN\right)-\left(FP \times FN\right)}{\sqrt{\left(TP+FP\right)\left(TP+FN\right)\left(TN+FP\right)\left(TN+FN\right)}} $$

Where, TP = True Positive, FP = False Positive, FN = False Negative, TN = True Negative.

## Abbreviations

AAC, Amino Acid Composition; DPC, dipeptide composition; FN, false negative; FP, false positive; GlcNAc, N-acetyl-D-glucosamine; mDAP, meso-diaminopimelate; MurNAc, N-Acetylmuramic acid; NAG, N-acetylglucosamine; NAM, β-(1–4)-N-acetylmuramic acid; OOB, out of bag; ORF, open reading frame; RF, Random Forest; SVM, Support Vector Machines; TN, true negative; TP, true positive.

## Additional files

Additional file 1: Performance evaluation of machine learning algorithms using WEKA on randomly selected test dataset for binary classification. (PDF 105 kb) Additional file 2: Performance evaluation of machine learning algorithms using WEKA on randomly selected test dataset for multiclass classification. (PDF 105 kb) Additional file 3: Categorization of peptidoglycan hydrolases into four different classes on the basis of their site of action and composition of Negative Dataset as the fifth class. (XLSX 8 kb) Additional file 4: Comparison of performance of the three approaches using known 250 peptidoglycan hydrolases. (XLSX 8 kb) Additional file 5: Comparison of performance of all the three approaches on a Metagenomic dataset. (XLSX 8 kb) Additional file 6: Performance of HyPe on independent genomic dataset. (XLSX 9 kb) Additional file 7: Metagenomic datasets used for evaluation of performance. (XLSX 9 kb) Additional file 8: Performance of HyPe on independent metagenomic dataset. (XLSX 9 kb) Additional file 9: The workflow of HyPe classification tool. (PDF 108 kb) Additional file 10: Instructions for running the stand-alone version of HyPe on the Linux PC. (TXT 845 bytes) Additional file 11: List of predicted peptidoglycan hydrolases from common pathogenic bacterial species. (PDF 734 kb) Additional file 12: Evaluation of domain based approach for the prediction of peptidoglycan hydrolases. (TXT 801 bytes)
