# Supplementary figures and images for: Prediction of peptidoglycan hydrolases- a new class of antibacterial proteins
Source: BMC Genomics. 2016 May 27;17:411. doi: 10.1186/s12864-016-2753-8 (PMC4882796; doi:10.1186/s12864-016-2753-8)

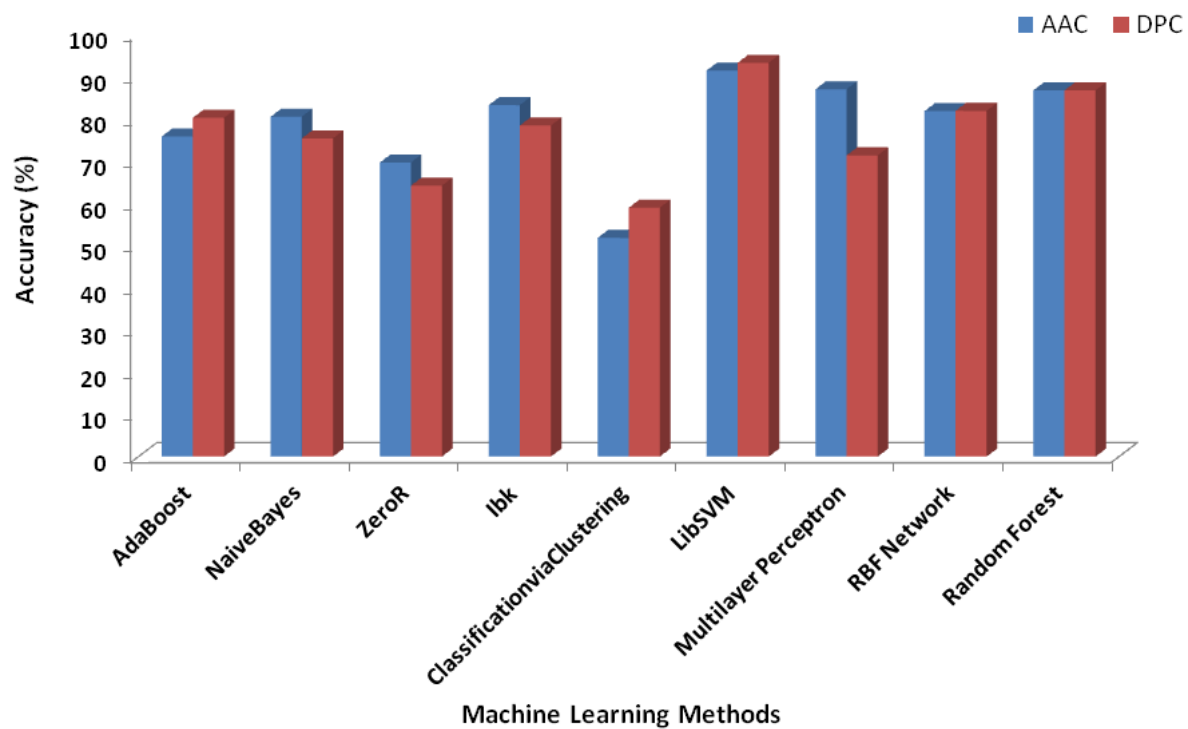

Supplement: Additional file 1: — Performance evaluation of machine learning algorithms using WEKA on randomly selected test dataset for binary classification. (PDF 105 kb) [file 12864_2016_2753_MOESM1_ESM.pdf]

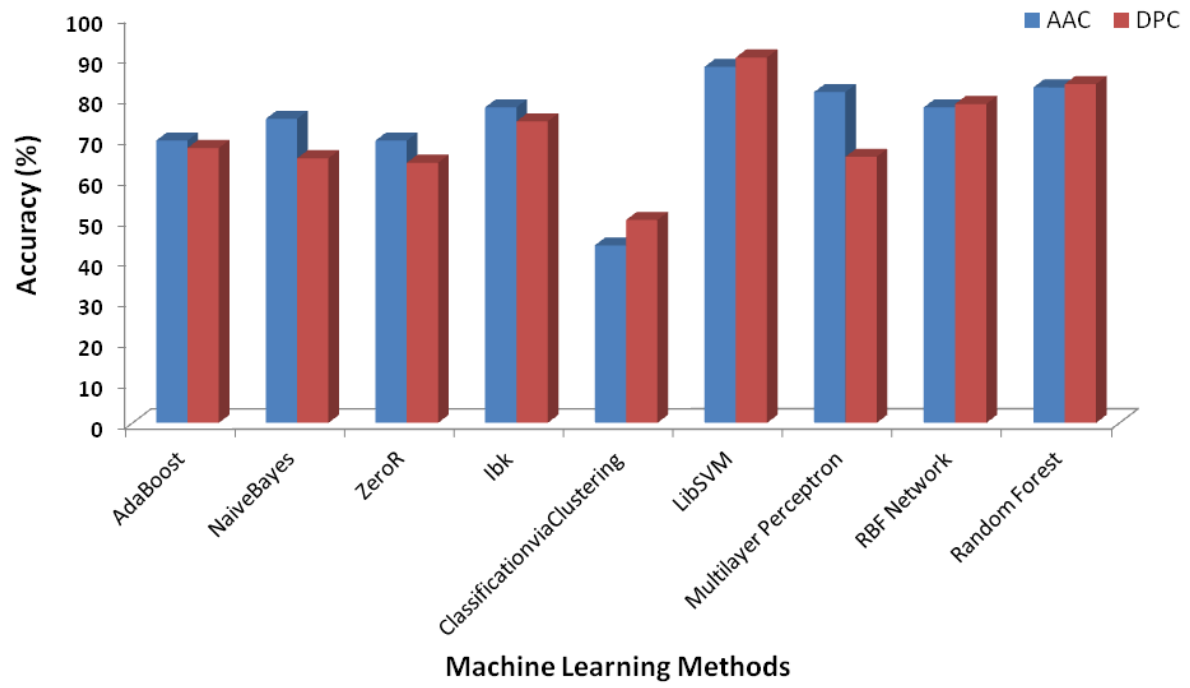

Supplement: Additional file 2: — Performance evaluation of machine learning algorithms using WEKA on randomly selected test dataset for multiclass classification. (PDF 105 kb) [file 12864_2016_2753_MOESM2_ESM.pdf]

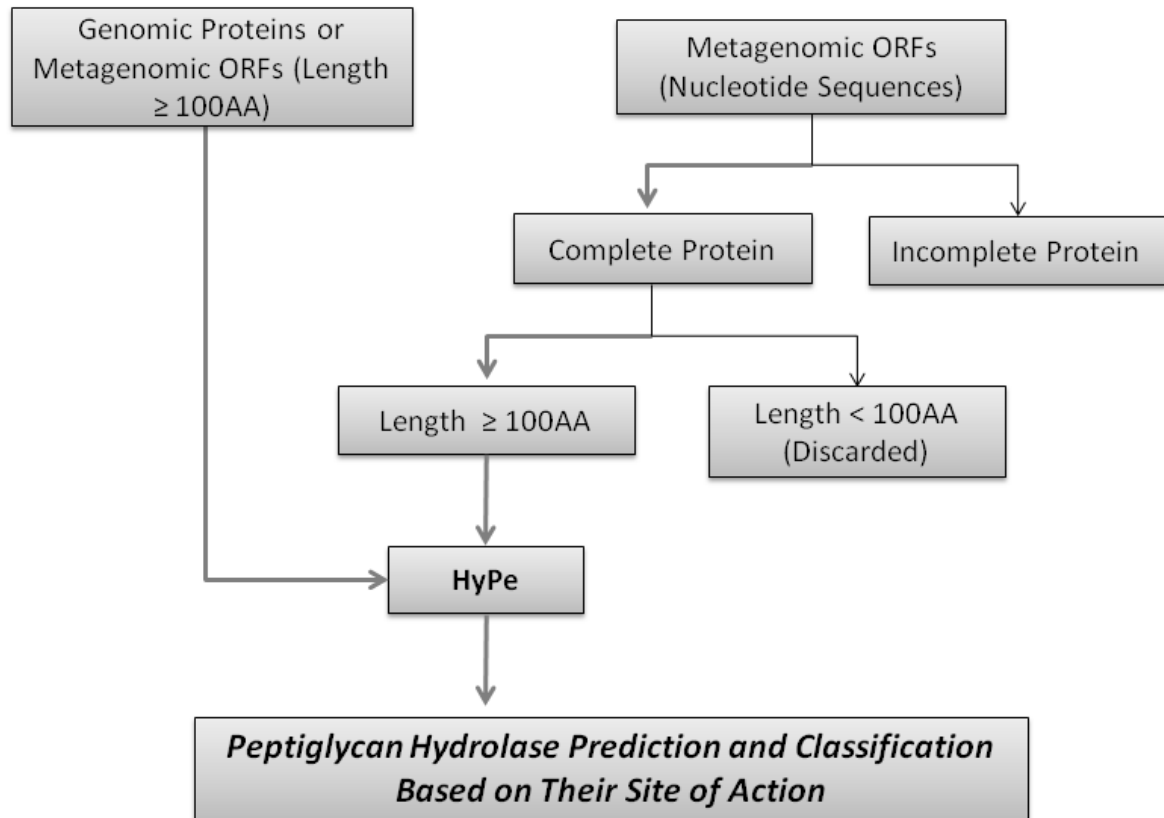

Supplement: Additional file 9: — The workflow of HyPe classification tool. (PDF 108 kb) [file 12864_2016_2753_MOESM9_ESM.pdf]
