# Supplementary material for: Prediction of peptidoglycan hydrolases- a new class of antibacterial proteins
Source: BMC Genomics. 2016 May 27;17:411. doi: 10.1186/s12864-016-2753-8 (PMC4882796; doi:10.1186/s12864-016-2753-8)
Supplement: Additional file 11: — List of predicted peptidoglycan hydrolases from common pathogenic bacterial species. (PDF 734 kb) [file 12864_2016_2753_MOESM11_ESM.pdf]

***Class: Predicted peptidoglycan hydrolase class**

Bacillus anthracis str. CDC 684		
**GI ID**	**Protein**	***Class**
gi|227812819|ref|YP_002812828.1	germination-specific N-acetylmuramoyl-L-alanine amidase	A
gi|227812888|ref|YP_002812897.1	invasion protein IagB domain protein	C
gi|227813153|ref|YP_002813162.1	hypothetical protein BAMEG_0551	A
gi|227813157|ref|YP_002813166.1	prophage LambdaBa02, N-acetylmuramoyl-L-alanine amidase PlyL	A
gi|227813464|ref|YP_002813473.1	prophage LambdaBa01, N-acetylmuramoyl-L-alanine amidase, family 2	A
gi|227813532|ref|YP_002813541.1	putative N-acetylmuramoyl-L-alanine amidase	A
gi|227813562|ref|YP_002813571.1	glycosyl hydrolase family protein	C
gi|227813638|ref|YP_002813647.1	hypothetical protein BAMEG_1042	A
gi|227813723|ref|YP_002813732.1	putative lysozyme	C
gi|227813783|ref|YP_002813792.1	bacillolysin	A
gi|227814330|ref|YP_002814339.1	x-prolyl-dipeptidyl aminopeptidase	A
gi|227814382|ref|YP_002814391.1	putative glycosyl hydrolase, family 25	A
gi|227814592|ref|YP_002814601.1	cell wall hydrolase	A
gi|227814662|ref|YP_002814671.1	N-acetylmuramoyl-L-alanine amidase family protein	A
gi|227814745|ref|YP_002814754.1	N-acetylmuramoyl-L-alanine amidase family protein	A
gi|227815025|ref|YP_002815034.1	hypothetical protein BAMEG_2436	A
gi|227815182|ref|YP_002815191.1	putative protease	B
gi|227815277|ref|YP_002815286.1	peptidase, M23/M37 family	A
gi|227815362|ref|YP_002815371.1	N-acetylmuramoyl-L-alanine amidase family protein	A
gi|227815467|ref|YP_002815476.1	transglycosylase	C
gi|227815659|ref|YP_002815668.1	N-acetylmuramoyl-L-alanine amidase family protein	A
gi|227816056|ref|YP_002816065.1	putative wall-associated protein	A
gi|227816163|ref|YP_002816172.1	putative S-layer protein	C
gi|227816237|ref|YP_002816246.1	surface-layer N-acetylmuramoyl-L-alanine amidase	A
gi|227816259|ref|YP_002816268.1	hypothetical protein BAMEG_3686	A
gi|227816529|ref|YP_002816538.1	neutral protease Npr599	A

gi|227816643|ref|YP_002816652.1	putative prophage LambdaBa04, glycosyl hydrolase, family 25	A
gi|227816649|ref|YP_002816658.1	hypothetical protein BAMEG_4127	A
gi|227817355|ref|YP_002817364.1	peptidase, M42 family	A
gi|227817953|ref|YP_002817962.1	putative cell wall endopeptidase, NlpC/P60 family	A
gi|227817995|ref|YP_002818004.1	putative cell wall hydrolase, partial	A
gi|227818158|ref|YP_002818167.1	cell wall hydrolase	A

Bacillus cereus ATCC 10987		
**GI ID**	**Protein**	***Class**
gi|42779227|ref|NP_976474.1	germination-specific N-acetylmuramoyl-L-alanine amidase	A
gi|42779321|ref|NP_976568.1	invasion protein IagB domain protein	C
gi|42779496|ref|NP_976743.1	N-acetylmuramoyl-L-alanine amidase domain protein	A
gi|42779745|ref|NP_976992.1	neutral protease	A
gi|42780060|ref|NP_977307.1	N-acetylmuramoyl-L-alanine amidase, family 3	A
gi|42780148|ref|NP_977395.1	S-layer protein, putative	C
gi|42780276|ref|NP_977523.1	wall associated protein, putative	A
gi|42780281|ref|NP_977528.1	cell wall-associated protein, putative	A
gi|42780349|ref|NP_977596.1	wall-associated protein, putative	A
gi|42780694|ref|NP_977941.1	N-acetylmuramoyl-L-alanine amidase, family 2	A
gi|42780831|ref|NP_978078.1	flagellar hook-associated protein, putative	A
gi|42780854|ref|NP_978101.1	transglycosylase, SLT family	C
gi|42780958|ref|NP_978205.1	N-acetylmuramoyl-L-alanine amidase, family 3	A
gi|42781022|ref|NP_978269.1	conserved hypothetical protein	A
gi|42781106|ref|NP_978353.1	NLP/P60 family protein	A
gi|42781139|ref|NP_978386.1	protease, putative	B
gi|42781473|ref|NP_978720.1	alkaline serine protease, subtilase family	A
gi|42781673|ref|NP_978920.1	cell wall hydrolase	A
gi|42781819|ref|NP_979066.1	neutral protease	A
gi|42781905|ref|NP_979152.1	peptidase, U32 family	A

gi|42781906|ref|NP_979153.1	N-acetylmuramoyl-L-alanine amidase	A
gi|42781947|ref|NP_979194.1	hydrolase, CocE/NonD family	A
gi|42782600|ref|NP_979847.1	conserved hypothetical protein	A
gi|42782677|ref|NP_979924.1	glycosyl hydrolase, family 18	C
gi|42782716|ref|NP_979963.1	N-acetylmuramoyl-L-alanine amidase, putative	A
gi|42782763|ref|NP_980010.1	glycosyl hydrolase, family 25, putative	A
gi|42783215|ref|NP_980462.1	5'-nucleotidase family protein	A
gi|42783748|ref|NP_980995.1	peptidase, M42 family	A
gi|42784346|ref|NP_981593.1	endopeptidase lytE, putative	A
gi|42784398|ref|NP_981645.1	enterotoxin	A
gi|42784564|ref|NP_981811.1	cell wall hydrolase	A
gi|16077111|ref|NP_387924.1	sporulation-specific protease YabG	B

Bacillus subtilis subsp. subtilis str. 168		
**GI ID**	**Protein**	***Class**
gi|16077221|ref|NP_388034.1	germination-specific N-acetylmuramoyl-L-alanine amidase	A
gi|255767073|ref|NP_388144.2	alkaline phosphatase D	A
gi|16077339|ref|NP_388152.1	lipase EstA	A
gi|16077350|ref|NP_388163.1	peptidoglycan L-alanyl-D-glutamate endopeptidase CwlK	B
gi|255767082|ref|NP_388186.2	alpha-amylase	A
gi|16077772|ref|NP_388586.1	rhamnogalacturonan endolyase YesW	A
gi|16077823|ref|NP_388637.1	pectate lyase	A
gi|16077851|ref|NP_388665.1	nucleotide phosphoesterase	A
gi|16078222|ref|NP_389039.1	murein lytic transglycosylase YjbJ	C
gi|16078311|ref|NP_389128.1	N-acetylmuramoyl-L-alanine amidase XlyB	A
gi|255767293|ref|NP_389150.2	phage-like element PBSX protein XkdO	C
gi|16078346|ref|NP_389164.1	N-acetylmuramoyl-L-alanine amidase XlyA	A
gi|16078534|ref|NP_389353.1	bacillolysin	A
gi|16078594|ref|NP_389413.1	bacillopeptidase F	A

gi|255767407|ref|NP_389623.2	sporulation-specific N-acetylmuramoyl-L-alanine amidase	A
gi|16078877|ref|NP_389698.1	arabinoxylan arabinofuranohydrolase	A
gi|16078896|ref|NP_389717.1	D-alanyl-D-alanine carboxypeptidase DacC	B
gi|255767473|ref|NP_390018.2	transglycosylase YomI	B
gi|16079200|ref|NP_390024.1	N-acetylmuramoyl-L-alanine amidase BlyA	A
gi|16079204|ref|NP_390028.1	glycosyltransferase SunS	C
gi|255767533|ref|NP_390299.2	hypothetical protein BSU24190	A
gi|16079624|ref|NP_390448.1	N-acetylmuramoyl-L-alanine amidase CwlH	A
gi|16079643|ref|NP_390467.1	N-acetylmuramoyl-L-alanine amidase CwlA	A
gi|255767599|ref|NP_390480.2	hypothetical protein BSU26030	C
gi|16079812|ref|NP_390636.1	N-acetylmuramoyl-L-alanine amidase YrvJ	A
gi|16080615|ref|NP_391442.1	N-acetylmuramoyl-L-alanine amidase LytC	A
gi|16080616|ref|NP_391443.1	amidase enhancer	A
gi|16080631|ref|NP_391459.1	beta-N-acetylglucosaminidase	C
gi|255767831|ref|NP_391802.2	wall-associated protein	A

Bacteroides fragilis 638R		
**GI ID**	**Protein**	***Class**
gi|375356633|ref|YP_005109405.1	putative lysozyme protein found in a conjugation transposase	C
gi|375356722|ref|YP_005109494.1	putative exported glutaminase	C
gi|375356725|ref|YP_005109497.1	putative TonB dependent receptor outer membrane protein	C
gi|375356759|ref|YP_005109531.1	putative TonB-dependent receptor exported protein	A
gi|375356781|ref|YP_005109553.1	putative TonB dependent outer membrane exported protein	C
gi|375356919|ref|YP_005109691.1	Putative TonB-linked outer membrane protein	A
gi|375356937|ref|YP_005109709.1	hypothetical protein BF638R_0567	C
gi|375357001|ref|YP_005109773.1	putative surface membrane protein	A
gi|375357003|ref|YP_005109775.1	putative TonB dependent outer membrane exported protein	A
gi|375357005|ref|YP_005109777.1	putative TonB dependent outer membrane protein	C
gi|375357029|ref|YP_005109801.1	hypothetical protein BF638R_0665	A

gi|375357098|ref|YP_005109870.1	putative lytic murein transglycosylase	C
gi|375357210|ref|YP_005109982.1	hypothetical protein BF638R_0862	C
gi|375357268|ref|YP_005110040.1	Putative TonB-dependent outer membrane receptor protein	A
gi|375357293|ref|YP_005110065.1	Putative TonB-dependent outer membrane receptor protein	A
gi|375357295|ref|YP_005110067.1	Putative TonB-dependent outer membrane receptor protein	A
gi|375357532|ref|YP_005110304.1	hypothetical protein BF638R_1208	C
gi|375357644|ref|YP_005110416.1	putative outer membrane protein	A
gi|375357817|ref|YP_005110589.1	putative hemagglutinin	A
gi|375358018|ref|YP_005110790.1	putative outer membrane protein	A
gi|375358024|ref|YP_005110796.1	putative TonB-dependent outer membrane receptor protein	A
gi|375358049|ref|YP_005110821.1	putative TonB-linked outer membrane protein	C
gi|375358277|ref|YP_005111049.1	putative bacteriophage Gp25 protein	C
gi|375358405|ref|YP_005111177.1	putative TonB-dependent outer membrane receptor protein	C
gi|375358406|ref|YP_005111178.1	hypothetical protein BF638R_2118	C
gi|375358443|ref|YP_005111215.1	putative TonB-dependent outer membrane receptor protein	C
gi|375358611|ref|YP_005111383.1	hypothetical protein BF638R_2330	A
gi|375358777|ref|YP_005111549.1	putative iron transport-related exported protein	C
gi|375358791|ref|YP_005111563.1	hypothetical protein BF638R_2515	A
gi|375358815|ref|YP_005111587.1	putative N-acetylmuramoyl-L-alanine amidase	A
gi|375359053|ref|YP_005111825.1	putative N-acetylmuramoyl-L-alanine amidase	A
gi|375359194|ref|YP_005111966.1	hypothetical protein BF638R_2948	A
gi|375359272|ref|YP_005112044.1	hypothetical protein BF638R_3031	A
gi|375359284|ref|YP_005112056.1	putative exported transglycosylase protein	C
gi|375359410|ref|YP_005112182.1	hypothetical protein BF638R_3170	A
gi|375359445|ref|YP_005112217.1	hypothetical protein BF638R_3206	C
gi|375359465|ref|YP_005112237.1	hypothetical protein BF638R_3226	C
gi|375359605|ref|YP_005112377.1	putative cell wall biosynthesis related protein	A
gi|375359621|ref|YP_005112393.1	putative peptidoglycan biosynthesis-related protein	C
gi|375359688|ref|YP_005112460.1	hypothetical protein BF638R_3466	A

gi|375359845|ref|YP_005112617.1	Putative N-acetylmuramoyl-L-alanine amidase	A
gi|375359849|ref|YP_005112621.1	hypothetical protein BF638R_3636	A
gi|375359899|ref|YP_005112671.1	putative exported N-acetylmuramoyl-L-alanine amidase	A
gi|375359996|ref|YP_005112768.1	hypothetical protein BF638R_3787	A
gi|375360294|ref|YP_005113066.1	putative outer membrane protein	A
gi|375360300|ref|YP_005113072.1	putative TonB-linked outer membrane protein	A
gi|375360411|ref|YP_005113183.1	hypothetical protein BF638R_4238	C
gi|375360478|ref|YP_005113250.1	hypothetical protein BF638R_4312	A
gi|375360497|ref|YP_005113269.1	putative outer membrane protein	A
gi|375360505|ref|YP_005113277.1	putative outer membrane protein	A
gi|375360602|ref|YP_005113374.1	Putative TonB-linked outer membrane protein	A

Bdellovibrio bacteriovorus HD100		
**GI ID**	**Protein**	***Class**
gi|42521787|ref|NP_967167.1	hypothetical protein Bd0150	C
gi|42521825|ref|NP_967205.1	hypothetical protein Bd0193	A
gi|42522132|ref|NP_967512.1	murein transglycosylase D	C
gi|42522311|ref|NP_967691.1	cell wall surface anchor family protein	A
gi|42522407|ref|NP_967787.1	adventurous gliding motility protein U	C
gi|42522455|ref|NP_967835.1	cell wall surface anchor family protein	A
gi|42522457|ref|NP_967837.1	hypothetical protein Bd0886	A
gi|42522591|ref|NP_967971.1	hypothetical protein Bd1032	B
gi|42522716|ref|NP_968096.1	hypothetical protein Bd1176	A
gi|42522774|ref|NP_968154.1	hypothetical protein Bd1247	A
gi|42522813|ref|NP_968193.1	fimbrial protein pilA	C
gi|42522854|ref|NP_968234.1	cell wall surface anchor family protein	A
gi|42522963|ref|NP_968343.1	cell wall surface anchor family protein	A
gi|42523042|ref|NP_968422.1	two-component hybrid sensor and regulator	A
gi|42523112|ref|NP_968492.1	molybdopterin oxidoreductase, iron-sulfur binding subunit	A

gi|42523126|ref|NP_968506.1	RTX family exoprotein	A
gi|42523208|ref|NP_968588.1	cell wall surface anchor family protein	C
gi|42523770|ref|NP_969150.1	subtilisin-like serine protease	A
gi|42523879|ref|NP_969259.1	cell wall surface anchor family protein	C
gi|42523970|ref|NP_969350.1	subtilisin like serine protease	A
gi|42523973|ref|NP_969353.1	hypothetical protein Bd2548	A
gi|42524003|ref|NP_969383.1	hypothetical protein Bd2581	A
gi|42524110|ref|NP_969490.1	protease	A
gi|42524117|ref|NP_969497.1	hypothetical protein Bd2699	A
gi|42524155|ref|NP_969535.1	hypothetical protein Bd2740	C
gi|42524282|ref|NP_969662.1	hemagglutinin/hemolysin-related protein	A
gi|42524640|ref|NP_970020.1	cell wall surface anchor family protein	C
gi|42524641|ref|NP_970021.1	cell wall surface anchor family protein	A
gi|42524780|ref|NP_970160.1	periplasmic binding transport protein	C

Bifidobacterium animalis subsp. lactis ATCC 27673		
**GI ID**	**Protein**	***Class**
gi|549471592|ref|YP_008605299.1	DO serine protease	A
gi|549472233|ref|YP_008605940.1	nicotinic acid mononucleotide adenylyltransferase	A
gi|549472416|ref|YP_008606123.1	hypothetical protein BLAC_04760	C
gi|549472492|ref|YP_008606199.1	N-acetyl transferase	C
gi|549472570|ref|YP_008606277.1	DNA gyrase subunit B	A
gi|549472623|ref|YP_008606330.1	oligopeptide ABC transporter periplasmic protein	A
gi|549472786|ref|YP_008606493.1	fibronectin type III	A
gi|549472820|ref|YP_008606527.1	hypothetical protein BLAC_06860	C
gi|549472826|ref|YP_008606533.1	dTDP-4-dehydrorhamnose 3,5-epimerase	A
gi|549472864|ref|YP_008606571.1	endo-1,4-beta-xylanase	A
gi|549472936|ref|YP_008606643.1	fimbrial subunit-like cell surface protein	A

gi|549472963|ref|YP_008606670.1	1,4-beta-N-acetylmuramidase	C
gi|549472987|ref|YP_008606694.1	xylan esterase	A
gi|549473045|ref|YP_008606752.1	hypothetical protein BLAC_08025	C

Bordetella pertussis CS		
**GI ID**	**Protein**	***Class**
gi|384202602|ref|YP_005588341.1	homoserine O-acetyltransferase	A
gi|384202624|ref|YP_005588363.1	N-acetyl-anhydromuranmyl-L-alanine amidase	A
gi|384202810|ref|YP_005588549.1	N-acetylmuramoyl-L-alanine amidase	A
gi|384202862|ref|YP_005588601.1	outer membrane heme receptor	C
gi|384202984|ref|YP_005588723.1	putative heme receptor	C
gi|384203218|ref|YP_005588957.1	putative ferric siderophore receptor	C
gi|384203308|ref|YP_005589047.1	outer membrane porin protein	C
gi|384203324|ref|YP_005589063.1	TonB-dependent receptor for iron transport	C
gi|384203325|ref|YP_005589064.1	TonB-dependent receptor for iron transport	C
gi|384203418|ref|YP_005589157.1	membrane-bound lytic murein transglycosylase B	C
gi|384203495|ref|YP_005589234.1	D-alanyl-D-alanine carboxypeptidase	B
gi|384203505|ref|YP_005589244.1	putative transglycosylase	C
gi|384203574|ref|YP_005589313.1	putative ferric siderophore receptor	C
gi|384203743|ref|YP_005589482.1	glycosyl hydrolase	C
gi|384203787|ref|YP_005589526.1	flagellar rod assembly protein/muramidase FlgJ	C
gi|384204113|ref|YP_005589852.1	putative peptidoglycan-binding protein	C
gi|384204127|ref|YP_005589866.1	hypothetical protein BPTD_1738	A
gi|384204229|ref|YP_005589968.1	hemolysin activator-like protein	C
gi|384204282|ref|YP_005590021.1	putative hemin storage protein	C
gi|384204297|ref|YP_005590036.1	putative ferrisiderophore receptor	C
gi|384204311|ref|YP_005590050.1	putative extracellular solute-binding protein	C
gi|384204340|ref|YP_005590079.1	putative lipoprotein	C
gi|384204341|ref|YP_005590080.1	putative ferric siderophore receptor	A

gi|384204360|ref|YP_005590099.1	amidase	C
gi|384204614|ref|YP_005590353.1	putative transferase	C
gi|384204679|ref|YP_005590418.1	putative binding-protein-dependent transport periplasmic protein	C
gi|384204728|ref|YP_005590467.1	putative exported hydrolase	A
gi|384204757|ref|YP_005590496.1	potassium-transporting ATPase subunit A	C
gi|384204928|ref|YP_005590667.1	phosphoribosylformylglycinamidine synthase	C
gi|384205087|ref|YP_005590826.1	membrane transport protein	C
gi|384205144|ref|YP_005590883.1	outer membrane receptor FepA	A
gi|384205150|ref|YP_005590889.1	adhesin	A
gi|384205165|ref|YP_005590904.1	putative TonB-dependent receptor	C
gi|384205222|ref|YP_005590961.1	histone protein	A
gi|384205244|ref|YP_005590983.1	putative D-amino acid dehydrogenase small subunit	C
gi|384205290|ref|YP_005591029.1	putative transglycosylase	C
gi|384205352|ref|YP_005591091.1	hypothetical protein BPTD_3090	C
gi|384205423|ref|YP_005591162.1	putative membrane-bound lytic murein transglycosylase D	C
gi|384205544|ref|YP_005591283.1	putative phage lysozyme	C
gi|384205566|ref|YP_005591305.1	hypothetical protein BPTD_3336	C
gi|384205666|ref|YP_005591405.1	serum resistance protein	A
gi|384205760|ref|YP_005591499.1	putative outer membrane protein	C
gi|384205824|ref|YP_005591563.1	ferric siderophore receptor	C

Borrelia burgdorferi **B31**		
**GI ID**	**Protein**	***Class**
gi|559797711|ref|YP_008853941.1	hypothetical protein BB_0531	A
gi|365992380|ref|NP_212759.2	N-acetylmuramoyl-L-alanine amidase	A
gi|365992389|ref|NP_212800.2	N-acetylmuramoyl-L-alanine amidase domain-containing protein	A

Brucella abortus S19		
**GI ID**	**Protein**	***Class**

gi|189023309|ref|YP_001934077.1	surface antigen	A
gi|189023327|ref|YP_001934095.1	SLT domain-containing protein	C
gi|189023374|ref|YP_001934142.1	outer membrane protein	C
gi|189023640|ref|YP_001934408.1	hypothetical protein BAbS19_I03950	C
gi|189023859|ref|YP_001934627.1	transglycosylase	C
gi|189024095|ref|YP_001934863.1	Zinc metalloprotease	C
gi|189024098|ref|YP_001934866.1	Cell wall hydrolase/autolysin	A
gi|189024153|ref|YP_001934921.1	Secretion activator protein	C
gi|189024205|ref|YP_001934973.1	Beta-lactamase	A
gi|189024215|ref|YP_001934983.1	ATP-dependent RNA helicase, DEAD/DEAH box family	A
gi|189024349|ref|YP_001935117.1	alkaline phosphatase	A
gi|189024549|ref|YP_001935317.1	hypothetical protein BAbS19_I13480	C
gi|189024567|ref|YP_001935335.1	SLT domain-containing protein	C
gi|189024568|ref|YP_001935336.1	peptidoglycan binding domain 1	A
gi|189024571|ref|YP_001935339.1	glycoside hydrolase family protein	C
gi|189024625|ref|YP_001935393.1	SH3 domain-containing protein	C
gi|189024844|ref|YP_001935612.1	peptidase family M48	A
gi|189024904|ref|YP_001935672.1	lytic murein transglycosylase	C
gi|189025075|ref|YP_001935843.1	outer membrane autotransporter	A

Brucella melitensis ATCC 23457		
**GI ID**	**Protein**	***Class**
gi|225851556|ref|YP_002731789.1	family 5 extracellular solute-binding protein	C
gi|225851590|ref|YP_002731823.1	surface antigen D15	A
gi|225851609|ref|YP_002731842.1	lytic transglycosylase	C
gi|225851658|ref|YP_002731891.1	porin	C
gi|225851938|ref|YP_002732171.1	hypothetical protein BMEA_A0433	C
gi|225852166|ref|YP_002732399.1	lytic transglycosylase	C
gi|225852422|ref|YP_002732655.1	peptidase M48 Ste24p	C

gi|225852426|ref|YP_002732659.1	N-acetylmuramoyl-L-alanine amidase	A
gi|225852490|ref|YP_002732723.1	hypothetical protein BMEA_A1023	C
gi|225852555|ref|YP_002732788.1	DEAD/DEAH box helicase	A
gi|225852696|ref|YP_002732929.1	alkaline phosphatase	A
gi|225852701|ref|YP_002732934.1	lytic transglycosylase	C
gi|225852904|ref|YP_002733137.1	outer membrane assembly lipoprotein YfiO	C
gi|225852924|ref|YP_002733157.1	lytic transglycosylase	C
gi|225852925|ref|YP_002733158.1	N-acetylmuramoyl-L-alanine amidase family 2 protein	A
gi|225852928|ref|YP_002733161.1	family 25 glycosyl hydrolase	C
gi|225853091|ref|YP_002733324.1	outer membrane protein Omp31	C
gi|225853211|ref|YP_002733444.1	peptidase M48 Ste24p	A
gi|225853270|ref|YP_002733503.1	lytic murein transglycosylase	C

Campylobacter jejuni subsp. jejuni NCTC 11168-BN148		
**GI ID**	**Protein**	***Class**
gi|403055418|ref|YP_006632823.1	secreted transglycosylase	C
gi|403055594|ref|YP_006632999.1	secreted transglycosylase	C
gi|403056004|ref|YP_006633409.1	N-acetylmuramoyl-L-alanine amidase	A
gi|403056192|ref|YP_006633597.1	flagellar hook-associated protein FlgK	A
gi|403056325|ref|YP_006633730.1	hemin uptake system outer membrane receptor	A
gi|403056387|ref|YP_006633792.1	lipoprotein	A
gi|403056437|ref|YP_006633842.1	flagellar hook protein FlgE	A

Chlamydia psittaci 01DC12		
**GI ID**	**Protein**	***Class**
gi|410858188|ref|YP_006974128.1	polymorphic outer membrane protein	A
gi|410858254|ref|YP_006974194.1	polymorphic outer membrane protein	A
gi|410858345|ref|YP_006974285.1	probable N-acetylmuramoyl-L-alanine amidase	A

gi|410858667|ref|YP_006974607.1	putative exported protein	C

Clostridium botulinum A str. ATCC 19397		
**GI ID**	**Protein**	***Class**
gi|153930851|ref|YP_001382608.1	spore-cortex-lytic enzyme	C
gi|153933510|ref|YP_001382836.1	N-acetylmuramoyl-L-alanine amidase-like protein	A
gi|153932683|ref|YP_001382895.1	NlpC/P60 family protein	A
gi|153932011|ref|YP_001383128.1	N-acetylmuramoyl-L-alanine amidase	A
gi|153933381|ref|YP_001383571.1	glycosyl hydrolase family protein	D
gi|153932511|ref|YP_001383745.1	hypothetical protein CLB_1418	C
gi|153931814|ref|YP_001383776.1	N-acetylmuramoyl-L-alanine amidase	A
gi|153933650|ref|YP_001383784.1	N-acetylmuramoyl-L-alanine amidase	A
gi|153932096|ref|YP_001384038.1	hypothetical protein CLB_1716	A
gi|153934162|ref|YP_001384040.1	mannosyl-glycoprotein endo-beta-N-acetylglucosaminidase domain-containing protein	C
gi|153933676|ref|YP_001384481.1	amidase	A
gi|153931921|ref|YP_001384731.1	N-acetylmuramoyl-L-alanine amidase	A
gi|153930828|ref|YP_001385112.1	N-acetylmuramoyl-L-alanine amidase	A
gi|153930977|ref|YP_001385246.1	N-acetylmuramoyl-L-alanine amidase	A
gi|153933559|ref|YP_001385331.1	transglycosylase	C
gi|153931252|ref|YP_001385335.1	N-acetylmuramoyl-L-alanine amidase	A
gi|153930900|ref|YP_001385432.1	N-acetylmuramoyl-L-alanine amidase	A
gi|153931954|ref|YP_001385433.1	N-acetylmuramoyl-L-alanine amidase	A
gi|153933593|ref|YP_001385435.1	beta-N-acetylglucosaminidase	C
gi|153933993|ref|YP_001385594.1	Slt family transglycosylase	C
gi|153932666|ref|YP_001385758.1	N-acetylmuramoyl-L-alanine amidase	A

Clostridium perfringens ATCC 13124		
**GI ID**	**Protein**	***Class**
gi|110799774|ref|YP_694575.1	N-acetylmuramoyl-L-alanine amidase	A

gi|110800117|ref|YP_695040.1	N-acetylmuramoyl-L-alanine amidase	A
gi|110799389|ref|YP_695058.1	cell wall binding repeat-containing protein/mannosyl-glycoprotein endo-beta-N-acetylglucosamidase domain-containing protein	A
gi|110801433|ref|YP_695416.1	KID repeat-containing protein	A
gi|110798593|ref|YP_695917.1	beta-galactosidase	A
gi|110799274|ref|YP_696005.1	NlpC/P60 family protein	A
gi|110800924|ref|YP_696189.1	N-acetylmuramoyl-L-alanine amidase	A
gi|110800912|ref|YP_696642.1	1A family penicillin-binding protein	A
gi|110800916|ref|YP_696671.1	Slt family transglycosylase	C
gi|110799813|ref|YP_697056.1	N-acetylmuramoyl-L-alanine amidase	A
gi|110800108|ref|YP_697250.1	serine protease C	A

Clostridium tetani 12124569		
**GI ID**	**Protein**	***Class**
gi|557604238|ref|YP_008772349.1	N-acetylmuramoyl-L-alanine amidase	A
gi|557604349|ref|YP_008772460.1	N-acetylmuramoyl-L-alanine amidase	A
gi|557604754|ref|YP_008772865.1	N-acetylmuramoyl-L-alanine amidase	A
gi|557605005|ref|YP_008773116.1	N-acetylmuramoyl-L-alanine amidase	A
gi|557605127|ref|YP_008773238.1	N-acetylmuramoyl-L-alanine amidase	A
gi|557605190|ref|YP_008773301.1	spore peptidoglycan hydrolase(N-acetylglucosaminidase)	D
gi|557605213|ref|YP_008773324.1	N-acetylmuramoyl-L-alanine amidase	A
gi|557605553|ref|YP_008773664.1	N-acetylmuramoyl-L-alanine amidase	A
gi|557605933|ref|YP_008774044.1	N-acetylmuramoyl-L-alanine amidase	A
gi|557606206|ref|YP_008774317.1	N-acetylmuramoyl-L-alanine amidase	A
gi|557606705|ref|YP_008774816.1	germination-specific N-acetylmuramoyl-L-alanineamidase	A

Corynebacterium diphtheriae CDCE 8392		
**GI ID**	**Protein**	***Class**
gi|376241875|ref|YP_005132727.1	N-acetylmuramoyl-L-alanine amidase	A

gi|376242098|ref|YP_005132950.1	putative secreted protein	C
gi|376242401|ref|YP_005133253.1	resuscitation-promoting factor A	C
gi|376242420|ref|YP_005133272.1	putative secreted protein	A
gi|376242512|ref|YP_005133364.1	putative secreted protein	C
gi|376242773|ref|YP_005133625.1	hypothetical protein CDCE8392_1084	A
gi|376243158|ref|YP_005134010.1	histidinol dehydrogenase	A
gi|376243213|ref|YP_005134065.1	hypothetical protein CDCE8392_1529	C
gi|376243216|ref|YP_005134068.1	ubiquinol-cytochrome C reductase cytochrome C subunit	C
gi|376243943|ref|YP_005134795.1	putative hydrolase	A

Coxiella burnetii RSA 331		
**GI ID**	**Protein**	***Class**
gi|161830043|ref|YP_001596320.1	N-acetylmuramoyl-L-alanine amidase/peptidoglycan binding domain-containing protein	A
gi|161830719|ref|YP_001596641.1	N-acetylmuramoyl-L-alanine amidase	A
gi|161830681|ref|YP_001596697.1	phage lysozyme	C
gi|161830665|ref|YP_001597802.1	D-alanyl-D-alanine carboxypeptidase/D-alanyl-D-alanine-endopeptidase	B

Deinococcus radiodurans R1		
**GI ID**	**Protein**	***Class**
gi|15805048|ref|NP_293732.1	metal-dependent hydrolase	A
gi|15805161|ref|NP_293847.1	hypothetical protein DR_0121	C
gi|15805407|ref|NP_294102.1	outer membrane protein	C
gi|15805410|ref|NP_294106.1	S-layer-like array-like protein	C
gi|15805432|ref|NP_294128.1	alpha-dextran endo-1,6-alpha-glucosidase	C
gi|15805588|ref|NP_294284.1	maltose ABC transporter periplasmic maltose-binding protein	C
gi|15805734|ref|NP_294430.1	hypothetical protein DR_0707	C
gi|15805735|ref|NP_294431.1	hypothetical protein DR_0708	C
gi|15805961|ref|NP_294661.1	TPR repeat-containing protein	C
gi|15806250|ref|NP_294955.1	hypothetical protein DR_1231	A

gi|15806308|ref|NP_295014.1	extracellular solute-binding protein	A
gi|15806404|ref|NP_295110.1	N-acetylmuramoyl-L-alanine amidase	A
gi|15806455|ref|NP_295161.1	ABC transporter periplasmic substrate-binding protein	C
gi|15806503|ref|NP_295214.1	carboxyl-terminal protease	C
gi|15806631|ref|NP_295348.1	amidase	A
gi|15806637|ref|NP_295355.1	N-acetylmuramoyl-L-alanine amidase	A
gi|15806891|ref|NP_295614.1	TPR repeat-containing protein	C
gi|15806953|ref|NP_295678.1	extracellular solute-binding protein	C
gi|15807248|ref|NP_295978.1	erythromycin esterase	C
gi|15807262|ref|NP_295992.1	hypothetical protein DR_2271	C
gi|15807301|ref|NP_296031.1	hypothetical protein DR_2310	C
gi|15807384|ref|NP_296115.1	N-acetylmuramoyl-L-alanine amidase	A
gi|15807493|ref|NP_296228.1	hexagonally packed intermediate-layer surface protein	A
gi|15807551|ref|NP_296287.1	N-acetylmuramoyl-L-alanine amidase-like protein	A
gi|15807560|ref|NP_296297.1	S-layer protein	A
gi|15807606|ref|NP_296345.1	carboxylesterase, type B	C

Ehrlichia canis **str. Jake**		
**GI ID**	**Protein**	***Class**
gi|73667469|ref|YP_303485.1	methionine aminopeptidase	A

Enterobacter cloacae subsp. cloacae ATCC 13047		
**GI ID**	**Protein**	***Class**
gi|296100391|ref|YP_003610537.1	maltose-6'-phosphate glucosidase	A
gi|296100417|ref|YP_003610563.1	putative maltoporin	C
gi|296100567|ref|YP_003610713.1	hypothetical protein ECL_00196	C
gi|296100595|ref|YP_003610741.1	putative lipase	C
gi|296100751|ref|YP_003610897.1	Putative RND efflux system, outer membrane lipoprotein	C
gi|296100783|ref|YP_003610929.1	pilus biogenesis protein PilL	A

gi|296100791|ref|YP_003610937.1	PilT protein	C
gi|296100798|ref|YP_003610944.1	hypothetical protein ECL_00429	C
gi|296100936|ref|YP_003611082.1	N-acetylmuramoyl-l-alanine amidase II	A
gi|296101055|ref|YP_003611201.1	putative transposase	C
gi|296101172|ref|YP_003611318.1	lytic murein transglycosylase	C
gi|296101272|ref|YP_003611418.1	anhydro-N-acetylmuramyl-tripeptide amidase	A
gi|296101287|ref|YP_003611433.1	Phage-related lysozyme (muraminidase)	C
gi|296101306|ref|YP_003611452.1	fimbrial usher protein	A
gi|296101376|ref|YP_003611522.1	membrane-bound lytic murein transglycosylase D	C
gi|296101383|ref|YP_003611529.1	hypothetical protein ECL_01017	A
gi|296101404|ref|YP_003611550.1	outer membrane efflux protein	C
gi|296101493|ref|YP_003611639.1	hypothetical protein ECL_01129	A
gi|296101683|ref|YP_003611829.1	phage lysozyme	C
gi|296101878|ref|YP_003612024.1	L,D-carboxypeptidase A	B
gi|296101879|ref|YP_003612025.1	lytic transglycosylase, catalytic	C
gi|296101882|ref|YP_003612028.1	TonB-dependent siderophore receptor	C
gi|296102071|ref|YP_003612217.1	gp16	C
gi|296102135|ref|YP_003612281.1	putative extracellular lipase	A
gi|296102159|ref|YP_003612305.1	murein peptide amidase A	B
gi|296102160|ref|YP_003612306.1	putative transport periplasmic protein	C
gi|296102224|ref|YP_003612370.1	TonB-dependent siderophore receptor	C
gi|296102430|ref|YP_003612576.1	outer membrane transport protein, type I secretion	C
gi|296102726|ref|YP_003612872.1	outer membrane usher protein LpfC	A
gi|296102769|ref|YP_003612915.1	hypothetical protein ECL_02423	C
gi|296102818|ref|YP_003612964.1	MltA-interacting MipA family protein	C
gi|296102880|ref|YP_003613026.1	ferric-rhodotorulic acid outer membrane transporter	C
gi|296102902|ref|YP_003613048.1	flagellar hook-associated protein FlgK	C
gi|296102903|ref|YP_003613049.1	flagellar rod assembly protein/muramidase FlgJ	C
gi|296103072|ref|YP_003613218.1	hypothetical protein ECL_02728	C

gi|296103130|ref|YP_003613276.1	hypothetical protein ECL_02786	C
gi|296103131|ref|YP_003613277.1	glycoside hydrolase family 18 protein	A
gi|296103148|ref|YP_003613294.1	N-acetylmuramoyl-L-alanine amidase	A
gi|296103230|ref|YP_003613376.1	peptide/nickel transport system substrate-binding protein	C
gi|296103255|ref|YP_003613401.1	outer membrane protein X	C
gi|296103346|ref|YP_003613492.1	alpha-ketoglutarate decarboxylase	A
gi|296103432|ref|YP_003613578.1	hypothetical protein ECL_03093	A
gi|296103510|ref|YP_003613656.1	TonB-dependent siderophore receptor	A
gi|296103546|ref|YP_003613692.1	phage lysozyme	C
gi|296103737|ref|YP_003613883.1	fimbrillin MatB	C
gi|296103782|ref|YP_003613928.1	D-alanyl-D-alanine endopeptidase	B
gi|296103833|ref|YP_003613979.1	putative peptide/nickel transport system substrate-binding protein	C
gi|296103909|ref|YP_003614055.1	phage lysozyme	C
gi|296104014|ref|YP_003614160.1	penicillin-insensitive murein endopeptidase	B
gi|296104095|ref|YP_003614241.1	N-acetylmuramoyl-l-alanine amidase I	A
gi|296104112|ref|YP_003614258.1	putative lipoprotein	C
gi|296104232|ref|YP_003614378.1	putative transglycosylase	C
gi|296104375|ref|YP_003614521.1	murein hydrolase B	C
gi|296104477|ref|YP_003614623.1	N-acetylmuramoyl-L-alanine amidase	A
gi|296104524|ref|YP_003614670.1	putative outer membrane protein A	C
gi|296104625|ref|YP_003614771.1	murein transglycosylase C	C
gi|296104689|ref|YP_003614835.1	iron complex outermembrane recepter protein	A
gi|296104696|ref|YP_003614842.1	hypothetical protein ECL_04364	C
gi|296104726|ref|YP_003614872.1	serine protease	C
gi|296104744|ref|YP_003614890.1	Phage baseplate assembly protein	C
gi|296104751|ref|YP_003614897.1	phage lysozyme	C
gi|296104893|ref|YP_003615039.1	D-alanyl-D-alanine carboxypeptidase	B
gi|296105248|ref|YP_003615394.1	hypothetical protein ECL_04921	C
gi|296105299|ref|YP_003615445.1	phospholipase A	C

gi|296105346|ref|YP_003615492.1	vitamin B12/cobalamin outer membrane transporter	A

Enterococcus faecalis 62		
**GI ID**	**Protein**	***Class**
gi|384516875|ref|YP_005704180.1	hypothetical protein EF62_0150	A
gi|384516984|ref|YP_005704289.1	hypothetical protein EF62_0259	A
gi|384517031|ref|YP_005704336.1	hypothetical protein EF62_0306	A
gi|384517244|ref|YP_005704549.1	nlpC/P60 family protein	B
gi|384518077|ref|YP_005705382.1	N-acetylmuramoyl-L-alanine amidase family protein	A
gi|384518197|ref|YP_005705502.1	Endocarditis and Biofilm-Associated Pilus subunitA	A
gi|384518333|ref|YP_005705638.1	extracellular solute-binding family protein	C
gi|384518390|ref|YP_005705695.1	N-acetylmuramoyl-L-alanine amidase family protein	A
gi|384518613|ref|YP_005705918.1	1,4-beta-N-acetylmuramoylhydrolase	C
gi|384518821|ref|YP_005706126.1	Ig-like domain (group 4) family protein	A
gi|384518887|ref|YP_005706192.1	NlpC/P60 family protein	B
gi|384519110|ref|YP_005706415.1	glycosyl hydrolases family 25 family protein	C
gi|384519152|ref|YP_005706457.1	extracellular solute-binding family protein	C
gi|384519185|ref|YP_005706490.1	internalin like repeat domain protein	C
gi|384519192|ref|YP_005706497.1	mannosyl-glycoprotein endo-beta-N-acetylglucosaminidase family protein	C
gi|384519428|ref|YP_005706733.1	mannosyl-glycoprotein endo-beta-N-acetylglucosaminidase autolysin family protein	A

Escherichia coli str. K-12 substr. DH10B		
**GI ID**	**Protein**	***Class**
gi|170079747|ref|YP_001729067.1	N-acetyl-anhydromuranmyl-L-alanine amidase	A
gi|170079842|ref|YP_001729162.1	membrane-bound lytic murein transglycosylase D	C
gi|170080136|ref|YP_001729456.1	DLP12 prophage; lysozyme	C
gi|170080137|ref|YP_001729457.1	DLP12 prophage; murein endopeptidase	B
gi|170080237|ref|YP_001729557.1	DLP12 prophage; lysozyme	C
gi|170080238|ref|YP_001729558.1	DLP12 prophage; murein endopeptidase	B

gi|170080464|ref|YP_001729784.1	catecholate siderophore receptor Fiu	A
gi|170080473|ref|YP_001729793.1	outer membrane protein X	C
gi|170080489|ref|YP_001729809.1	periplasmic solute-binding component of a glutathione transporter	C
gi|170080526|ref|YP_001729846.1	amidase	A
gi|170080583|ref|YP_001729903.1	hypothetical protein ECDH10B_0995	C
gi|170080681|ref|YP_001730001.1	outer membrane protein PgaA	C
gi|170080732|ref|YP_001730052.1	flagellar rod assembly protein/muramidase FlgJ	C
gi|170080733|ref|YP_001730053.1	flagellar hook-associated protein FlgK	C
gi|170080806|ref|YP_001730126.1	hypothetical protein ECDH10B_1231	C
gi|170080820|ref|YP_001730140.1	L,D-carboxypeptidase A	B
gi|170080830|ref|YP_001730150.1	adhesin	A
gi|170080903|ref|YP_001730223.1	lysozyme-like protein	C
gi|170080973|ref|YP_001730293.1	antibiotic ABC transporter periplasmic-binding protein	C
gi|170081004|ref|YP_001730324.1	murein peptide amidase A	A
gi|170081008|ref|YP_001730328.1	L-ala-gamma-D-glutamyl-meso-DAP ABC transporter periplasmic substrate-binding protein	C
gi|170081049|ref|YP_001730369.1	Rac prophage; tail fiber protein	A
gi|170081166|ref|YP_001730486.1	porin protein	A
gi|170081177|ref|YP_001730497.1	hypothetical protein ECDH10B_1640	A
gi|170081178|ref|YP_001730498.1	putative lipoprotein	A
gi|170081222|ref|YP_001730542.1	Qin prophage; lysozyme	C
gi|170081379|ref|YP_001730699.1	hypothetical protein ECDH10B_1860	C
gi|170081629|ref|YP_001730949.1	CP4-44 prophage; antigen 43 (Ag43) phase-variable biofilm formation autotransporter	A
gi|170081727|ref|YP_001731047.1	hydrolase	C
gi|170081733|ref|YP_001731053.1	outer membrane protein	A
gi|170081757|ref|YP_001731077.1	D-alanyl-D-alanine endopeptidase	B
gi|170081835|ref|YP_001731155.1	outer membrane porin protein C	C
gi|170081851|ref|YP_001731171.1	adhesin	A
gi|170081943|ref|YP_001731263.1	hypothetical protein ECDH10B_2489	B
gi|170081944|ref|YP_001731264.1	penicillin-insensitive murein endopeptidase	B

gi|170081960|ref|YP_001731280.1	long-chain fatty acid outer membrane transporter	C
gi|170082044|ref|YP_001731364.1	N-acetylmuramoyl-l-alanine amidase I	A
gi|170082104|ref|YP_001731424.1	peptidase	C
gi|170082168|ref|YP_001731488.1	putative transglycosylase	C
gi|170082252|ref|YP_001731572.1	LysM domain/BON superfamily protein	C
gi|170082277|ref|YP_001731597.1	murein hydrolase B	C
gi|170082385|ref|YP_001731705.1	N-acetylmuramoyl-L-alanine amidase	A
gi|170082515|ref|YP_001731835.1	murein transglycosylase C	C
gi|170082580|ref|YP_001731900.1	hypothetical protein ECDH10B_3211	C
gi|170082681|ref|YP_001732001.1	outer membrane protein	A
gi|170082717|ref|YP_001732037.1	D-alanyl-D-alanine carboxypeptidase	B
gi|170082742|ref|YP_001732062.1	monofunctional biosynthetic peptidoglycan transglycosylase	C
gi|170082844|ref|YP_001732164.1	general secretory pathway component, cryptic	A
gi|170083040|ref|YP_001732360.1	cellulose synthase subunit BcsC	C
gi|170083079|ref|YP_001732399.1	hypothetical protein ECDH10B_3751	C
gi|170083435|ref|YP_001732755.1	vitamin B12/cobalamin outer membrane transporter	A
gi|170083472|ref|YP_001732792.1	isocitrate lyase	A
gi|170083600|ref|YP_001732920.1	beta-lactamase	B
gi|170083615|ref|YP_001732935.1	N-acetylmuramoyl-l-alanine amidase II	A
gi|170083746|ref|YP_001733066.1	N-acetylnuraminic acid outer membrane channel protein	C
gi|170083778|ref|YP_001733098.1	lytic murein transglycosylase	C

Eubacterium rectale ATCC 33656		
**GI ID**	**Protein**	***Class**
gi|238922455|ref|YP_002935968.1	germination-specific N-acetylmuramoyl-L-alanine amidase	A
gi|238922889|ref|YP_002936402.1	galactose/glucose-binding lipoprotein	A
gi|238922938|ref|YP_002936451.1	hypothetical protein EUBREC_0526	A
gi|238923299|ref|YP_002936814.1	cell surface protein	C
gi|238923300|ref|YP_002936815.1	N-acetylmuramoyl-L-alanine amidase	A

gi|238923349|ref|YP_002936864.1	N-acetylmuramoyl-L-alanine amidase domain protein	A
gi|238923461|ref|YP_002936977.1	periplasmic alpha-amylase precursor	C
gi|238923496|ref|YP_002937012.1	hypothetical protein EUBREC_1116	A
gi|238923511|ref|YP_002937027.1	hypothetical protein in ImpB/MucB/SamB family of UV repair proteins	A
gi|238923828|ref|YP_002937344.1	soluble lytic murein transglycosylase precursor	C
gi|238924227|ref|YP_002937743.1	hypothetical protein EUBREC_1865	A
gi|238924298|ref|YP_002937814.1	N-acetylmuramoyl-L-alanine amidase, C-terminus, partial	A
gi|238924347|ref|YP_002937863.1	sporulation protein SpoIID	A
gi|238924438|ref|YP_002937954.1	glycoside hydrolase family 25	C
gi|238924445|ref|YP_002937961.1	hypothetical protein EUBREC_2086	C
gi|238924531|ref|YP_002938047.1	hypothetical protein EUBREC_2173	A
gi|238924660|ref|YP_002938176.1	mannosyl-glycoprotein endo-beta-N-acetylglucosaminidase	C
gi|238924728|ref|YP_002938244.1	N-acetylmuramoyl-L-alanine amidase	A
gi|238924984|ref|YP_002938500.1	N-acetylmuramoyl-L-alanine amidase	A
gi|238924985|ref|YP_002938501.1	glycoside hydrolase family 25	A
gi|238925098|ref|YP_002938615.1	cysteine proteinase	A
gi|238925103|ref|YP_002938620.1	cellulose 1,4-beta-cellobiosidase	A
gi|238925600|ref|YP_002939117.1	flagellar capping protein	C
gi|238925642|ref|YP_002939159.1	glycoside hydrolase family 25	C
gi|238926005|ref|YP_002939523.1	hypothetical protein EUBREC_3663	A
Francisella tularensis subsp. tularensis FSC198		
**GI ID**	**Protein**	***Class**
gi|110669802|ref|YP_666359.1	N-acetyl-anhydromuranmyl-L-alanine amidase	A
gi|110670020|ref|YP_666577.1	soluble lytic murein transglycosylase	C
gi|110670080|ref|YP_666637.1	organic solvent tolerance protein	C
gi|110670142|ref|YP_666699.1	lipoprotein	C
gi|110670305|ref|YP_666862.1	chitinase family 18 protein	C
gi|110670597|ref|YP_667154.1	D-alanyl-D-alanine carboxypeptidase	B

gi|110670598|ref|YP_667155.1	lipoprotein	C
gi|110670844|ref|YP_667401.1	inosine-5-monophosphate dehydrogenase	A

Haemophilus influenzae R2846		
**GI ID**	**Protein**	***Class**
gi|386265241|ref|YP_005828733.1	Putative penicillin-binding protein 7	B
gi|386265303|ref|YP_005828795.1	N-acetyl-anhydromuramyl-L-alanine amidase AmpD	A
gi|386265372|ref|YP_005828864.1	Adhesion and penetration protein precursor	A
gi|386265385|ref|YP_005828877.1	membrane-bound lytic murein transglycosylase F	C
gi|386265444|ref|YP_005828936.1	Putative permease YcfA	B
gi|386265445|ref|YP_005828937.1	murein DD-endopeptidase, penicillin-insensitive	B
gi|386265564|ref|YP_005829056.1	N-acetylmuramoyl-L-alanine amidase	A
gi|386265598|ref|YP_005829090.1	Penicillin-binding protein 2	A
gi|386265714|ref|YP_005829206.1	Lipoprotein LppC	C
gi|386265913|ref|YP_005829405.1	D-alanyl-D-alanine carboxypeptidase/D-alanyl-D-alanine-endopeptidase	B
gi|386266274|ref|YP_005829766.1	Immunoglobulin A1 protease	A
gi|386266507|ref|YP_005829999.1	Membrane-bound lytic murein transglycosylase C	C
gi|386266561|ref|YP_005830053.1	Outer membrane antigenic lipoprotein B	C
gi|386266608|ref|YP_005830100.1	Hemoglobin and hemoglobin-haptoglobin binding protein B	A

Helicobacter pylori 52		
**GI ID**	**Protein**	***Class**
gi|384887064|ref|YP_005761575.1	outer membrane protein HopD	C
gi|384887254|ref|YP_005761765.1	outer membrane protein (omp29)	C
gi|384887315|ref|YP_005761826.1	putative vacuolating cytotoxin (VacA)-like protein	A
gi|384887360|ref|YP_005761871.1	hypothetical protein HPKB_0346	C
gi|384887581|ref|YP_005762092.1	N-acetylmuramoyl-L-alanine amidase	A
gi|384887698|ref|YP_005762209.1	transglycosylase	C
gi|384887732|ref|YP_005762243.1	putative vacuolating cytotoxin (VacA)	A

gi|384888054|ref|YP_005762565.1	outer membrane protein HopI	C
gi|384888132|ref|YP_005762643.1	outer membrane protein - adhesin	A
gi|384888357|ref|YP_005762868.1	iron-regulated outer membrane protein	A
gi|384888380|ref|YP_005762891.1	TonB-dependent siderophore receptor	A
gi|384888435|ref|YP_005762946.1	lytic transglycosylase, catalytic	C

Klebsiella pneumoniae subsp. pneumoniae 1084		
**GI ID**	**Protein**	***Class**
gi|402778318|ref|YP_006633864.1	maltose-6'-phosphate glucosidase	A
gi|402778476|ref|YP_006634022.1	BAX protein	C
gi|402778520|ref|YP_006634066.1	cellulose synthase operon protein C	C
gi|402778809|ref|YP_006634355.1	D-alanyl-D-alanine carboxypeptidase	B
gi|402778872|ref|YP_006634418.1	Pirin-like protein YhaK	A
gi|402778960|ref|YP_006634506.1	type I secretion outer membrane protein, TolC	C
gi|402779007|ref|YP_006634553.1	membrane-bound lytic murein transglycosylase C	C
gi|402779101|ref|YP_006634647.1	type 1 fimbriae anchoring protein FimD	A
gi|402779170|ref|YP_006634716.1	N-acetylmuramoyl-L-alanine amidase	A
gi|402779292|ref|YP_006634838.1	TonB-dependent hemin , ferrichrome receptor	C
gi|402779344|ref|YP_006634890.1	membrane-bound lytic murein transglycosylase B	C
gi|402779365|ref|YP_006634911.1	L-proline glycine betaine binding ABC transporter protein ProX	C
gi|402779394|ref|YP_006634940.1	type 1 fimbriae anchoring protein FimD	C
gi|402779493|ref|YP_006635039.1	Slt family transglycosylase	C
gi|402779563|ref|YP_006635109.1	outer membrane protein NlpB	C
gi|402779596|ref|YP_006635142.1	N-acetylmuramoyl-L-alanine amidase	A
gi|402779642|ref|YP_006635188.1	long-chain fatty acid transport protein	C
gi|402779649|ref|YP_006635195.1	murein endopeptidase	B
gi|402779734|ref|YP_006635280.1	outer membrane protein C	C
gi|402779759|ref|YP_006635305.1	ABC transporter substrate-binding protein	C
gi|402779798|ref|YP_006635344.1	murein-DD-endopeptidase	B

gi|402780059|ref|YP_006635605.1	outer membrane protein N	C
gi|402780096|ref|YP_006635642.1	alpha,alpha-trehalose-phosphate synthase	C
gi|402780185|ref|YP_006635731.1	muramoyltetrapeptide carboxypeptidase	B
gi|402780242|ref|YP_006635788.1	hypothetical protein A79E_1987	C
gi|402780310|ref|YP_006635856.1	outer membrane protein	C
gi|402780392|ref|YP_006635938.1	ferrichrome-iron receptor	A
gi|402780409|ref|YP_006635955.1	ABC transporter, periplasmmic iron binding protein	C
gi|402780590|ref|YP_006636136.1	tonB-dependent receptor yncD	C
gi|402781035|ref|YP_006636581.1	outer membrane protein N	C
gi|402781101|ref|YP_006636647.1	hypothetical protein A79E_2852	C
gi|402781117|ref|YP_006636663.1	Periplasmic Murein Peptide-Binding Protein MppA	C
gi|402781118|ref|YP_006636664.1	Gamma-D-Glutamyl-meso-Diaminopimelate Amidase	B
gi|402781185|ref|YP_006636731.1	ferrichrome-iron receptor	A
gi|402781299|ref|YP_006636845.1	phage tail fiber protein	C
gi|402781476|ref|YP_006637022.1	Phytanoyl-CoA dioxygenase	A
gi|402781586|ref|YP_006637132.1	N-acetylmuramoyl-L-alanine amidase	A
gi|402781623|ref|YP_006637169.1	dipeptide-binding ABC transporter, periplasmic substrate-binding component	C
gi|402781644|ref|YP_006637190.1	Attachment invasion locus protein	C
gi|402781806|ref|YP_006637352.1	Penicillin-binding protein 2 (PBP-2)	A
gi|402781907|ref|YP_006637453.1	GntR family transcriptional regulator domain / Aspartate aminotransferase	A
gi|402782080|ref|YP_006637626.1	maltoporin	C
gi|402782294|ref|YP_006637840.1	membrane-bound lytic murein transglycosylase D	C
gi|402782409|ref|YP_006637955.1	N-acetylmuramoyl-L-alanine amidase	A
gi|402782523|ref|YP_006638069.1	lytic murein transglycosylase	C
gi|402782681|ref|YP_006638227.1	lytic murein transglycosylase	C
gi|402782837|ref|YP_006638383.1	N-acetylmuramoyl-L-alanine amidase	A
gi|402782951|ref|YP_006638497.1	Slt family transglycosylase	C
gi|402783183|ref|YP_006638729.1	formate dehydrogenase O subunit alpha	A

Lactococcus lactis subsp. lactis KLDS 4.0325		
**GI ID**	**Protein**	***Class**
gi|554464938|ref|YP_008701786.1	hypothetical protein P620_01430	A
gi|554463348|ref|YP_008701923.1	ABC transporter substrate-binding protein	C
gi|554463473|ref|YP_008702091.1	penicillin-binding protein 1A	C
gi|554465057|ref|YP_008702289.1	hypothetical protein P620_04160	A
gi|554465066|ref|YP_008702374.1	hypothetical protein P620_04600	A
gi|554465140|ref|YP_008702577.1	hypothetical protein P620_05650	A
gi|554465254|ref|YP_008702802.1	hypothetical protein P620_06795	C
gi|554465335|ref|YP_008702995.1	N-acetyl-muramidase	C
gi|554465384|ref|YP_008703125.1	cell-wall-anchored protein SasA (LPXTG motif)	C
gi|554465389|ref|YP_008703158.1	hypothetical protein P620_08610	A
gi|554464258|ref|YP_008703299.1	transglycosylase	C
gi|554465505|ref|YP_008703562.1	hypothetical protein P620_10695	A
gi|554465507|ref|YP_008703564.1	hypothetical protein P620_10705	D
gi|554464492|ref|YP_008703639.1	N-acetylmuramoyl-L-alanine amidase	A
gi|554465584|ref|YP_008703859.1	hypothetical protein P620_12275	A

Leptospira interrogans serovar Lai str. 56601		
**GI ID**	**Protein**	***Class**
gi|24213159|ref|NP_710640.1	N-acetylmuramoyl-L-alanine amidase	A
gi|294827714|ref|NP_710890.2	hypothetical protein LA_0709	A
gi|24213491|ref|NP_710972.1	transglycosylase	C
gi|24214269|ref|NP_711750.1	hypothetical protein LA_1569	A
gi|24214900|ref|NP_712381.1	amidase	A
gi|24216032|ref|NP_713513.1	cytoplasmic membrane protein	A
gi|294828308|ref|NP_713576.2	hypothetical protein LA_3396	A
gi|24216133|ref|NP_713614.1	N-acetylmuramoyl-L-alanine amidase	A
gi|24216424|ref|NP_713905.1	cytoplasmic membrane protein	A

gi|24216569|ref|NP_714050.1	hypothetical protein LA_3870	A
gi|24216971|ref|NP_714452.1	hypothetical protein LA_4272	C

Listeria monocytogenes ATCC 19117		
**GI ID**	**Protein**	***Class**
gi|405748350|ref|YP_006671816.1	cell wall surface anchor family protein	A
gi|405748480|ref|YP_006671946.1	N-acetylmuramoyl-L-alanine amidase	A
gi|405748481|ref|YP_006671947.1	Ser/Thr protein phosphatase family protein	A
gi|405748794|ref|YP_006672260.1	WapA family wall-associated protein	A
gi|405748840|ref|YP_006672306.1	hypothetical protein LMOATCC19117_0505	A
gi|405748993|ref|YP_006672459.1	cell wall surface anchor family protein	C
gi|405749088|ref|YP_006672554.1	transglycosylase	C
gi|405749430|ref|YP_006672896.1	N-acetylmuramoyl-L-alanine amidase	A
gi|405749549|ref|YP_006673015.1	N-acetylmuramoyl-L-alanine amidase	A
gi|405749550|ref|YP_006673016.1	N-acetylmuramoyl-L-alanine amidase	A
gi|405749865|ref|YP_006673331.1	N-acetylmuramoyl-L-alanine amidase	A
gi|405750016|ref|YP_006673482.1	cell wall surface anchor family protein	A
gi|405750297|ref|YP_006673763.1	cell wall surface anchor family protein	A
gi|405750563|ref|YP_006674029.1	N-acetylmuramoyl-L-alanine amidase	A
gi|405750788|ref|YP_006674254.1	glycosyl hydrolase family protein	A
gi|405750902|ref|YP_006674368.1	N-acetylmuramoyl-L-alanine amidase	A
gi|405750936|ref|YP_006674402.1	N-acetylmuramoyl-L-alanine amidase	A
gi|405751035|ref|YP_006674501.1	N-acetylmuramoyl-L-alanine amidase	A

Mycobacterium tuberculosis H37Rv		
**GI ID**	**Protein**	***Class**
gi|397671843|ref|YP_006513377.1	oxidoreductase	A
gi|397672251|ref|YP_006513786.1	prolyl oligopeptidase	A
gi|397672479|ref|YP_006514014.1	lipoprotein LpqP	A

gi|397672489|ref|YP_006514024.1	transcriptional regulator TetR-family	C
gi|397672548|ref|YP_006514083.1	hypothetical protein RVBD_0739	C
gi|397672755|ref|YP_006514290.1	periplasmic phosphate-binding lipoprotein PstS1	C
gi|397672848|ref|YP_006514383.1	lipoprotein LpqU	C
gi|397673070|ref|YP_006514605.1	membrane protein	C
gi|397673091|ref|YP_006514626.1	hypothetical protein RVBD_1251c	A
gi|397673128|ref|YP_006514663.1	mycolyltransferase II	A
gi|397673555|ref|YP_006515090.1	outer membrane protein MctB	A
gi|397673613|ref|YP_006515148.1	fatty-acid-CoA ligase FadD1	C
gi|397673617|ref|YP_006515152.1	hypothetical protein RVBD_1754c	C
gi|397673701|ref|YP_006515236.1	hypothetical protein RVBD_1836c	A
gi|397673817|ref|YP_006515352.1	hypothetical protein RVBD_1949c	C
gi|397673890|ref|YP_006515425.1	transposase	C
gi|397674020|ref|YP_006515555.1	lipoprotein LppL	A
gi|397674216|ref|YP_006515751.1	periplasmic sugar-binding lipoprotein UspC	C
gi|397674448|ref|YP_006515983.1	hypothetical protein RVBD_2542	A
gi|397674493|ref|YP_006516028.1	protein-export membrane protein SecD	C
gi|397674892|ref|YP_006516427.1	exported or membrane protein	A
gi|397674983|ref|YP_006516518.1	short-chain alcohol dehydrogenase/reductase	A
gi|397675382|ref|YP_006516917.1	PPE family protein PPE59	C
gi|397675391|ref|YP_006516926.1	transmembrane protein	C
gi|397675493|ref|YP_006517028.1	PPE family protein PPE63	C
gi|397675548|ref|YP_006517083.1	lipoprotein LpqF	A
gi|397675549|ref|YP_006517084.1	hypothetical protein RVBD_3594	A
gi|397675581|ref|YP_006517116.1	d-alanyl-d-alanine carboxypeptidase	B
gi|397675674|ref|YP_006517209.1	N-acetylmuramoyl-L-alanine amidase	A
gi|397675772|ref|YP_006517308.1	hypothetical protein RVBD_3811	A
gi|397675860|ref|YP_006517396.1	hypothetical protein RVBD_3896c	C
gi|397675880|ref|YP_006517416.1	N-acetylmuramoyl-L-alanine amidase	A

Myxococcus xanthus DK 1622		
**GI ID**	**Protein**	***Class**
gi|108757004|ref|YP_628299.1	PHB depolymerase esterase	A
gi|108757154|ref|YP_628397.1	transglycosylase SLT domain-containing protein	C
gi|108762740|ref|YP_628492.1	transglycosylase SLT domain-containing protein	C
gi|108757512|ref|YP_628501.1	hypothetical protein MXAN_0219	C
gi|108762055|ref|YP_628619.1	N-acetylmuramoyl-L-alanine amidase	A
gi|108760456|ref|YP_628832.1	LysM domain-containing protein	A
gi|108759659|ref|YP_629243.1	hypothetical protein MXAN_0982	A
gi|108758072|ref|YP_629248.1	N-acetylmuramoyl-L-alanine amidase	A
gi|108762289|ref|YP_629281.1	hypothetical protein MXAN_1020	A
gi|108758609|ref|YP_629452.1	hypothetical protein MXAN_1193	C
gi|108757613|ref|YP_629924.1	hypothetical protein MXAN_1672	C
gi|108761612|ref|YP_629983.1	cysteine-rich repeat-containing protein	A
gi|108758052|ref|YP_630238.1	N-acetylmuramoyl-L-alanine amidase	A
gi|108761197|ref|YP_630373.1	beta-lactamase	C
gi|108758669|ref|YP_630480.1	hypothetical protein MXAN_2259	C
gi|108759519|ref|YP_630535.1	RIO1 family protein	A
gi|108757922|ref|YP_631031.1	N-acetylmuramoyl-L-alanine amidase	A
gi|108758904|ref|YP_631087.1	hypothetical protein MXAN_2876	A
gi|108758326|ref|YP_631147.1	hypothetical protein MXAN_2937	A
gi|108762908|ref|YP_631284.1	transglycosylase SLT domain-containing protein	C
gi|108758082|ref|YP_631332.1	S9A family peptidase	A
gi|108758656|ref|YP_631560.1	transglycosylase SLT domain-containing protein	C
gi|108759376|ref|YP_631754.1	M36 family peptidase	A
gi|108763549|ref|YP_631756.1	amidase	C
gi|108761464|ref|YP_631843.1	hypothetical protein MXAN_3656	A
gi|108759818|ref|YP_631863.1	hypothetical protein MXAN_3676	C

gi|108757251|ref|YP_632066.1	N-acetylmuramoyl-L-alanine amidase	A
gi|108762638|ref|YP_632179.1	N-acetylmuramoyl-L-alanine amidase	A
gi|108763880|ref|YP_632213.1	transglycosylase SLT domain-containing protein	C
gi|108759662|ref|YP_632309.1	hypothetical protein MXAN_4134	C
gi|108759224|ref|YP_632450.1	hypothetical protein MXAN_4275	C
gi|108757180|ref|YP_632494.1	metallo-beta-lactamase	A
gi|108762752|ref|YP_632562.1	catalase KatB	A
gi|108761453|ref|YP_632794.1	transglycosylase SLT domain-containing protein	C
gi|108761241|ref|YP_632826.1	pilus biogenesis protein	C
gi|108762121|ref|YP_632998.1	endoglucanase	C
gi|108761653|ref|YP_633010.1	hypothetical protein MXAN_4849, partial	A
gi|108758494|ref|YP_633421.1	lysyl endopeptidase domain-containing protein	C
gi|108762709|ref|YP_633476.1	phytase	C
gi|108762400|ref|YP_633530.1	lipoprotein	A
gi|108759536|ref|YP_633538.1	hypothetical protein MXAN_5391	A
gi|108758096|ref|YP_633597.1	M36 family peptidase	C
gi|108761320|ref|YP_633974.1	Ig-like domain-containing protein	A
gi|108758420|ref|YP_634267.1	Ig-like domain/kelch domain-containing protein	C
gi|108761497|ref|YP_634374.1	kelch motif-containing protein	A
gi|108758856|ref|YP_634494.1	transglycosylase SLT domain-containing protein	C
gi|108757507|ref|YP_634574.1	beta-lactamase	C
gi|108761912|ref|YP_634796.1	hypothetical protein MXAN_6679	A
gi|108761913|ref|YP_634817.1	hypothetical protein MXAN_6700	C
gi|108759598|ref|YP_635219.1	Tat pathway signal sequence domain-containing protein	A
gi|108759605|ref|YP_635431.1	Slt family transglycosylase	D
gi|108761372|ref|YP_635494.1	class I chitinase	C

Neisseria meningitidis G2136		
**GI ID**	**Protein**	***Class**

gi|385339376|ref|YP_005893248.1	TonB-dependent siderophore receptor	C
gi|385339471|ref|YP_005893343.1	hemagglutinin/hemolysin family protein	A
gi|385339546|ref|YP_005893418.1	copper ABC transporter periplasmic copper-binding protein	A
gi|385339593|ref|YP_005893465.1	putative putrescene ABC transporter, periplasmic putrescine-binding protein	C
gi|385339625|ref|YP_005893497.1	hypothetical protein NMBG2136_0596	A
gi|385339644|ref|YP_005893516.1	N-acetylmuramoyl-L-alanine amidase	A
gi|385339676|ref|YP_005893548.1	IgA-specific serine endopeptidase	A
gi|385339727|ref|YP_005893599.1	D-alanyl-D-alanine carboxypeptidase	B
gi|385339976|ref|YP_005893848.1	N-acetylmuramoyl-L-alanine amidase	A
gi|385340003|ref|YP_005893875.1	hypothetical protein NMBG2136_0990	C
gi|385340010|ref|YP_005893882.1	sulfate ABC transporter substrate-binding protein	C
gi|385340206|ref|YP_005894078.1	transglycosylase SLT/LysM domain protein	C
gi|385340352|ref|YP_005894224.1	transglycosylase SLT domain-containing protein	C
gi|385340369|ref|YP_005894241.1	LysM domain/M23 peptidase domain protein	C
gi|385340377|ref|YP_005894249.1	heme-utilization protein Hup	A
gi|385340416|ref|YP_005894288.1	lactoferrin-binding protein A	C
gi|385340490|ref|YP_005894362.1	hypothetical protein NMBG2136_1505	C
gi|385340613|ref|YP_005894485.1	transferrin-binding protein 1	C
gi|385340618|ref|YP_005894490.1	N-acetylmuramoyl-L-alanine amidase	A
gi|385340695|ref|YP_005894567.1	hypothetical protein NMBG2136_1726	A
gi|385340795|ref|YP_005894667.1	transglycosylase SLT domain-containing protein	C
gi|385340816|ref|YP_005894688.1	hypothetical protein NMBG2136_1862	C
gi|385340818|ref|YP_005894690.1	hemoglobin and hemoglobin-haptoglobin-binding protein B precursor	C

Paenibacillus polymyxa CR1		
**GI ID**	**Protein**	***Class**
gi|565990188|ref|YP_008909728.1	pectate lyase	A
gi|565990534|ref|YP_008910104.1	peptidase m28	A
gi|565990569|ref|YP_008910141.1	hypothetical protein X809_03145	A

gi|565990822|ref|YP_008910410.1	rhamnogalacturonate lyase	A
gi|565990877|ref|YP_008910469.1	hypothetical protein X809_04870	A
gi|565990989|ref|YP_008910587.1	phospholipid phosphatase	C
gi|565991163|ref|YP_008910803.1	hypothetical protein X809_06555	C
gi|565991166|ref|YP_008910807.1	N-acetylmuramoyl-L-alanine amidase	A
gi|565991466|ref|YP_008911122.1	beta-xylosidase	C
gi|565991557|ref|YP_008911220.1	lytic transglycosylase	C
gi|565994995|ref|YP_008911279.1	hypothetical protein X809_09020	A
gi|565991611|ref|YP_008911280.1	type IV secretion protein Rhs	A
gi|565991688|ref|YP_008911358.1	N-acetylmuramoyl-L-alanine amidase	A
gi|565995051|ref|YP_008912089.1	hypothetical protein X809_13220	C
gi|565992414|ref|YP_008912160.1	lytic transglycosylase	C
gi|565992438|ref|YP_008912195.1	arabinoxylan arabinofuranohydrolase	A
gi|565992703|ref|YP_008912489.1	cwlv	A
gi|565992704|ref|YP_008912490.1	cwlu	A
gi|565992727|ref|YP_008912514.1	alkaline phosphatase	A
gi|565992919|ref|YP_008912710.1	DNA-directed RNA polymerase subunit beta	A
gi|565993024|ref|YP_008912818.1	sugar ABC transporter	C
gi|565993151|ref|YP_008912948.1	lytic transglycosylase	C
gi|565993417|ref|YP_008913258.1	TldD protein	A
gi|565993466|ref|YP_008913326.1	serine protease	A
gi|565993481|ref|YP_008913341.1	endo-1,3-beta-glucanase	A
gi|565993784|ref|YP_008913657.1	bacillolysin	A
gi|565993785|ref|YP_008913658.1	bacillolysin	A
gi|565993839|ref|YP_008913714.1	mannanase	A
gi|565994167|ref|YP_008914070.1	N-acetylmuramoyl-L-alanine amidase	A
gi|565994303|ref|YP_008914243.1	mannan endo-1,4-beta-mannosidase	A
gi|565994371|ref|YP_008914322.1	copper amine oxidase	C
gi|565994375|ref|YP_008914326.1	hypothetical protein X809_25035	A

gi|565994592|ref|YP_008914555.1	arabinanase	A
gi|565994639|ref|YP_008914606.1	alpha-amylase	A
gi|565994659|ref|YP_008914626.1	hypothetical protein X809_26560	C
gi|565994809|ref|YP_008914794.1	cellulase	A
Pasteurella multocida subsp. multocida str. 3480		
**GI ID**	**Protein**	***Class**
gi|386833945|ref|YP_006239260.1	AmiC protein	A
gi|386834140|ref|YP_006239455.1	D-alanyl-D-alanine carboxypeptidase/D-alanyl-D-alanine-endopeptidase	B
gi|386834231|ref|YP_006239546.1	LppC	C
gi|386834525|ref|YP_006239841.1	peptidoglycan amidase MepA	B
gi|386834817|ref|YP_006240134.1	N-acetylmuramyl L-alanine amydase, putative	A
gi|386834871|ref|YP_006240188.1	phage endolysin, putative	C
gi|386834909|ref|YP_006240226.1	ArtI protein	C
gi|386834954|ref|YP_006240271.1	2',3'-cyclic-nucleotide 2'-phosphodiesterase	A
gi|386835445|ref|YP_006240764.1	MapA protein	A
gi|386835501|ref|YP_006240820.1	HasR protein	C
gi|386835696|ref|YP_006241016.1	hypothetical protein	A

Prevotella ruminicola 23		
**GI ID**	**Protein**	***Class**
gi|294672897|ref|YP_003573513.1	N-acetylmuramoyl-L-alanine amidase	A
gi|294672936|ref|YP_003573552.1	TonB dependent receptor	A
gi|294673066|ref|YP_003573682.1	hypothetical protein PRU_0294	C
gi|294673151|ref|YP_003573767.1	transglycosylase SLT domain-containing protein	C
gi|294673377|ref|YP_003573993.1	outer membrane receptor (OMR) family transporter	A
gi|294673527|ref|YP_003574143.1	N-acetylmuramoyl-L-alanine amidase	A
gi|294673552|ref|YP_003574168.1	hypothetical protein PRU_0814	C
gi|294673698|ref|YP_003574314.1	hemagglutinin	A

gi|294673742|ref|YP_003574358.1	hypothetical protein PRU_1026	C
gi|294673796|ref|YP_003574412.1	outer membrane receptor (OMR) family transporter	A
gi|294673840|ref|YP_003574456.1	N-acetylmuramoyl-L-alanine amidase	A
gi|294673864|ref|YP_003574480.1	TonB dependent receptor	A
gi|294673865|ref|YP_003574481.1	lipoprotein	C
gi|294673928|ref|YP_003574544.1	lipoprotein	A
gi|294673950|ref|YP_003574566.1	hypothetical protein PRU_1251	A
gi|294673966|ref|YP_003574582.1	TonB dependent receptor	A
gi|294673969|ref|YP_003574585.1	GLUG domain-containing protein	A
gi|294673984|ref|YP_003574600.1	N-acetylmuramoyl-L-alanine amidase	A
gi|294674152|ref|YP_003574768.1	outer membrane protein SusC	A
gi|294674153|ref|YP_003574769.1	hypothetical protein PRU_1463	C
gi|294674254|ref|YP_003574870.1	lipoprotein	A
gi|294674258|ref|YP_003574874.1	N-acetylmuramoyl-L-alanine amidase	A
gi|294674270|ref|YP_003574886.1	hypothetical protein PRU_1586	C
gi|294674274|ref|YP_003574890.1	receptor antigen RagA	A
gi|294674278|ref|YP_003574894.1	outer membrane protein SusC	A
gi|294674453|ref|YP_003575069.1	N-acetylmuramoyl-L-alanine amidase	A
gi|294674508|ref|YP_003575124.1	RHS/YD repeat protein	C
gi|294674515|ref|YP_003575131.1	N-acetylmuramoyl-L-alanine amidase	A
gi|294674542|ref|YP_003575158.1	receptor antigen RagA	A
gi|294674543|ref|YP_003575159.1	lipoprotein	C
gi|294674544|ref|YP_003575160.1	lipoprotein	C
gi|294674551|ref|YP_003575167.1	lysozyme	C
gi|294674554|ref|YP_003575170.1	hypothetical protein PRU_1886	A
gi|294674555|ref|YP_003575171.1	lipoprotein	C
gi|294674556|ref|YP_003575172.1	TonB dependent receptor	A
gi|294674557|ref|YP_003575173.1	hypothetical protein PRU_1889	C
gi|294674614|ref|YP_003575230.1	receptor antigen RagA	A

<table>
<tr><td>gi|294674730|ref|YP_003575346.1</td><td>hypothetical protein PRU_2074</td><td>A</td></tr>
<tr><td>gi|294674732|ref|YP_003575348.1</td><td>family 43 glycosyl hydrolase</td><td>A</td></tr>
<tr><td>gi|294674819|ref|YP_003575435.1</td><td>hypothetical protein PRU_2169</td><td>C</td></tr>
<tr><td>gi|294674823|ref|YP_003575439.1</td><td>hypothetical protein PRU_2173</td><td>A</td></tr>
<tr><td>gi|294674869|ref|YP_003575485.1</td><td>lipoprotein</td><td>C</td></tr>
<tr><td>gi|294674870|ref|YP_003575486.1</td><td>TonB dependent receptor</td><td>A</td></tr>
<tr><td>gi|294674873|ref|YP_003575489.1</td><td>hypothetical protein PRU_2228</td><td>A</td></tr>
<tr><td>gi|294674914|ref|YP_003575530.1</td><td>hypothetical protein PRU_2272</td><td>A</td></tr>
<tr><td>gi|294675062|ref|YP_003575678.1</td><td>N-acetylmuramoyl-L-alanine amidase</td><td>A</td></tr>
<tr><td>gi|294675072|ref|YP_003575688.1</td><td>outer membrane protein</td><td>A</td></tr>
<tr><td>gi|294675073|ref|YP_003575689.1</td><td>lipoprotein</td><td>C</td></tr>
<tr><td>gi|294675079|ref|YP_003575695.1</td><td>receptor antigen RagA</td><td>A</td></tr>
<tr><td>gi|294675122|ref|YP_003575738.1</td><td>outer membrane receptor (OMR) family transporter</td><td>A</td></tr>
<tr><td>gi|294675312|ref|YP_003575928.1</td><td>outer membrane protein SusC</td><td>A</td></tr>
<tr><td>gi|294675328|ref|YP_003575944.1</td><td>hypothetical protein PRU_2697</td><td>A</td></tr>
<tr><td>gi|294675335|ref|YP_003575951.1</td><td>TonB dependent receptor</td><td>A</td></tr>
<tr><td>gi|294675363|ref|YP_003575979.1</td><td>hypothetical protein PRU_2734</td><td>A</td></tr>
<tr><td>gi|294675365|ref|YP_003575981.1</td><td>hypothetical protein PRU_2736</td><td>A</td></tr>
<tr><td>gi|294675429|ref|YP_003576045.1</td><td>lipoprotein</td><td>A</td></tr>
<tr><td>gi|294675448|ref|YP_003576064.1</td><td>receptor antigen RagA</td><td>A</td></tr>
<tr><td>gi|294675517|ref|YP_003576133.1</td><td>hypothetical protein PRU_2899</td><td>C</td></tr>
</table>

<table>
<tr><td>Pseudomonas aeruginosa B136-33</td><td></td><td></td></tr>
<tr><th>GI ID</th><th>Protein</th><th>*Class</th></tr>
<tr><td>gi|478476228|ref|YP_007706879.1</td><td>phospholipase C</td><td>A</td></tr>
<tr><td>gi|478476251|ref|YP_007706902.1</td><td>hypothetical protein G655_00255</td><td>A</td></tr>
<tr><td>gi|478476289|ref|YP_007706940.1</td><td>hypothetical protein G655_00445</td><td>C</td></tr>
<tr><td>gi|478476391|ref|YP_007707042.1</td><td>putative TonB-dependent receptor</td><td>C</td></tr>
<tr><td>gi|478476588|ref|YP_007707239.1</td><td>hypothetical protein G655_01950</td><td>C</td></tr>
</table>

gi|478476776|ref|YP_007707427.1	hypothetical protein G655_02890	C
gi|478476822|ref|YP_007707473.1	putative bacteriophage protein	C
gi|478476825|ref|YP_007707476.1	tail fiber	C
gi|478476835|ref|YP_007707486.1	lytic enzyme	C
gi|478476955|ref|YP_007707606.1	putative porin	C
gi|478477131|ref|YP_007707782.1	penicillin-binding protein 2	A
gi|478477133|ref|YP_007707784.1	lytic murein transglycosylase B	C
gi|478477141|ref|YP_007707792.1	putative murein transglycosylase	C
gi|478477236|ref|YP_007707887.1	Fe(III) dicitrate transport protein FecA	C
gi|478477303|ref|YP_007707954.1	Rhs family protein-like protein	C
gi|478477391|ref|YP_007708042.1	membrane-bound lytic transglycosylase F	C
gi|478477623|ref|YP_007708274.1	putative serine protease	C
gi|478477718|ref|YP_007708369.1	hypothetical protein G655_07610	C
gi|478477736|ref|YP_007708387.1	secretion protein	C
gi|478477835|ref|YP_007708486.1	phosphonate ABC transporter periplasmic phosphonate-binding protein	C
gi|478478073|ref|YP_007708724.1	glycine betaine transmethylase	A
gi|478478074|ref|YP_007708725.1	hypothetical protein G655_09390	C
gi|478478106|ref|YP_007708757.1	D-alanyl-D-alanine carboxypeptidase/D-alanyl-D-alanine-endopeptidase	B
gi|478478133|ref|YP_007708784.1	putative soluble lytic transglycosylase	C
gi|478478246|ref|YP_007708897.1	TonB-dependent receptor	A
gi|478478292|ref|YP_007708943.1	Slt family transglycosylase	C
gi|478478743|ref|YP_007709394.1	hypothetical protein G655_12755	C
gi|478478782|ref|YP_007709433.1	porin	C
gi|478478824|ref|YP_007709475.1	hypothetical protein G655_13160	C
gi|478478841|ref|YP_007709492.1	type VI secretion protein IcmF	C
gi|478478867|ref|YP_007709518.1	TonB-dependent receptor	C
gi|478478928|ref|YP_007709579.1	penicillin-binding protein 3A	A
gi|478478971|ref|YP_007709622.1	hypothetical protein G655_13895	C
gi|478479123|ref|YP_007709774.1	TonB-dependent receptor	C

gi|478479142|ref|YP_007709793.1	TonB dependent receptor	C
gi|478479155|ref|YP_007709806.1	hypothetical protein G655_14815	C
gi|478479177|ref|YP_007709828.1	thiamine pyrophosphate protein	C
gi|478479345|ref|YP_007709996.1	LasA protease	C
gi|478479408|ref|YP_007710059.1	membrane-bound lytic murein transglycosylase D	C
gi|478479567|ref|YP_007710218.1	hypothetical protein G655_16890	C
gi|478479951|ref|YP_007710602.1	putative tonB-dependent receptor	A
gi|478480067|ref|YP_007710718.1	transglycosylase	C
gi|478480158|ref|YP_007710809.1	flagellar hook-associated protein FlgK	C
gi|478480228|ref|YP_007710879.1	cis,cis-muconate transporter MucK	C
gi|478480243|ref|YP_007710894.1	hypothetical protein G655_20275	C
gi|478480389|ref|YP_007711040.1	D-alanyl-D-alanine endopeptidase	B
gi|478480407|ref|YP_007711058.1	chitin-binding protein CbpD	C
gi|478480452|ref|YP_007711103.1	AmpDh3	A
gi|478480571|ref|YP_007711222.1	hypothetical protein G655_21915	A
gi|478480758|ref|YP_007711409.1	murein hydrolase B	C
gi|478480814|ref|YP_007711465.1	ABC transporter binding protein	C
gi|478480816|ref|YP_007711467.1	binding protein component of ABC transporter	C
gi|478480836|ref|YP_007711487.1	N-acetyl-anhydromuranmyl-L-alanine amidase	A
gi|478480914|ref|YP_007711565.1	soluble lytic murein transglycosylase	C
gi|478480986|ref|YP_007711637.1	type 4 fimbrial biogenesis protein	C
gi|478481045|ref|YP_007711696.1	catalase	A
gi|478481083|ref|YP_007711734.1	hypothetical protein G655_24520	C
gi|478481145|ref|YP_007711796.1	Heme/Hemoglobin uptake outer membrane receptor PhuR precursor	A
gi|478481312|ref|YP_007711963.1	hypothetical protein G655_25665	C
gi|478481344|ref|YP_007711995.1	putative extracellular heme-binding protein	C
gi|478481395|ref|YP_007712046.1	N-acetylmuramoyl-L-alanine amidase	A
gi|478481422|ref|YP_007712073.1	outer membrane protein	C
gi|478481480|ref|YP_007712131.1	short chain dehydrogenase	C

gi|478481652|ref|YP_007712303.1	LD-carboxypeptidase	B
gi|478481776|ref|YP_007712427.1	putative binding protein component of ABC dipeptide transporter	C
gi|478481946|ref|YP_007712597.1	N-acetylmuramoyl-L-alanine amidase	A
gi|478481998|ref|YP_007712649.1	N-acetylmuramoyl-L-alanine amidase	A

Rickettsia rickettsii str. Hino		
**GI ID**	**Protein**	***Class**
gi|379017678|ref|YP_005293913.1	negative regulator of beta-lactamase expression	A
gi|379017733|ref|YP_005293968.1	soluble lytic murein transglycosylase	C
gi|379018235|ref|YP_005294470.1	outer membrane protein B	A
gi|379018490|ref|YP_005294725.1	N-acetylmuramoyl-L-alanine amidase	A

Salmonella enterica subsp. enterica serovar Typhi str. CT18		
**GI ID**	**Protein**	***Class**
gi|16759009|ref|NP_454626.1	hypothetical protein STY0016	C
gi|16759143|ref|NP_454760.1	anhydro-N-acetylmuramyl-tripeptide amidase	A
gi|16759246|ref|NP_454863.1	membrane-bound lytic murein transglycosylase D	C
gi|16759255|ref|NP_454872.1	type VI system protein	C
gi|16759284|ref|NP_454901.1	Rhs-family protein	C
gi|16759351|ref|NP_454968.1	autotransporter/virulence factor	A
gi|16759751|ref|NP_455368.1	outer membrane protein X	C
gi|16759766|ref|NP_455383.1	ABC transporter substrate-binding protein	C
gi|16759804|ref|NP_455421.1	N-acetylmuramoyl-L-alanine amidase	A
gi|16759815|ref|NP_455432.1	macrolide transporter ATP-binding protein/permease	A
gi|16759864|ref|NP_455481.1	murein L,D-transpeptidase	C
gi|16759903|ref|NP_455520.1	bacteriophage protein	C
gi|16759933|ref|NP_455550.1	hypothetical protein STY1072	C
gi|16760058|ref|NP_455675.1	flagellar protein FlgJ	C

gi|16760059|ref|NP_455676.1	flagellar hook-associated protein 1	C
gi|16760207|ref|NP_455824.1	murein peptide amidase A	A
gi|16760208|ref|NP_455825.1	periplasmic murein peptide-binding protein MppA	C
gi|16760258|ref|NP_455875.1	glucan biosynthesis protein D	C
gi|16760292|ref|NP_455909.1	TonB-dependent receptor YncD	A
gi|16760428|ref|NP_456045.1	bacteriophage baseplate protein	C
gi|16760442|ref|NP_456059.1	outer membrane protein	C
gi|16760608|ref|NP_456225.1	outer membrane protein	C
gi|16760660|ref|NP_456277.1	hypothetical protein STY1889	C
gi|16760690|ref|NP_456307.1	L,D-carboxypeptidase A	B
gi|16760757|ref|NP_456374.1	bacteriophage tail protein	C
gi|16760787|ref|NP_456404.1	endolysin	C
gi|16760937|ref|NP_456554.1	outer membrane protein S1	C
gi|16761307|ref|NP_456924.1	penicillin-insensitive murein endopeptidase	B
gi|16761368|ref|NP_456985.1	N-acetylmuramoyl-L-alanine amidase	A
gi|16761395|ref|NP_457012.1	esterase	A
gi|16761429|ref|NP_457046.1	exodeoxyribonuclease large subunit	A
gi|16761431|ref|NP_457048.1	hypothetical protein STY2760	A
gi|16761541|ref|NP_457158.1	large repetitive protein	A
gi|16761607|ref|NP_457224.1	membrane-bound lytic transglycosylase B	C
gi|16761654|ref|NP_457271.1	cell invasion protein	D
gi|16761767|ref|NP_457384.1	N-acetylmuramoyl-L-alanine amidase	A
gi|16761888|ref|NP_457505.1	membrane-bound lytic murein transglycosylase C	C
gi|16762062|ref|NP_457679.1	penicillin-binding protein	B
gi|16762256|ref|NP_457873.1	lysozyme	C
gi|16762262|ref|NP_457879.1	phage baseplate assembly protein	C
gi|16762647|ref|NP_458264.1	exported amidase	C
gi|16762664|ref|NP_458281.1	biotin sulfoxide reductase	A
gi|16762667|ref|NP_458284.1	hypothetical protein STY4161	A

gi|16762697|ref|NP_458314.1	phage-like lysozyme	C
gi|16763038|ref|NP_458655.1	hypothetical protein STY4570	A
gi|16763079|ref|NP_458696.1	phage baseplate assembly protein	C
gi|16763084|ref|NP_458701.1	hypothetical protein STY4619	C
gi|16763085|ref|NP_458702.1	lysozyme	C
gi|16763177|ref|NP_458794.1	N-acetylmuramoyl-L-alanine amidase	A
gi|541470449|ref|NP_458990.3	lytic murein transglycosylase	C

Salmonella enterica subsp. enterica serovar Typhimurium str. DT104		
**GI ID**	**Protein**	***Class**
gi|550899447|ref|YP_008668539.1	hypothetical protein DT104_00151	C
gi|550899571|ref|YP_008668663.1	anhydro-N-acetylmuramyl-tripeptide amidase	A
gi|550899675|ref|YP_008668767.1	membrane-bound lytic murein transglycosylase D	C
gi|550899682|ref|YP_008668774.1	type vi secretion system lysozyme-like SciD protein	C
gi|550899687|ref|YP_008668779.1	type IV secretion system SciI protein	C
gi|550899707|ref|YP_008668799.1	rhs-family protein	C
gi|550899761|ref|YP_008668853.1	Endopeptidase	B
gi|550899827|ref|YP_008668919.1	autotransporter/virulence factor	A
gi|550900249|ref|YP_008669341.1	outer membrane protein X precursor	C
gi|550900264|ref|YP_008669356.1	ABC transporter substrate-binding protein	C
gi|550900307|ref|YP_008669399.1	N-acetylmuramoyl-L-alanine amidase	A
gi|550900318|ref|YP_008669410.1	ABC transporter	A
gi|550900369|ref|YP_008669461.1	hypothetical protein DT104_09691	C
gi|550900404|ref|YP_008669496.1	phage lysozyme	C
gi|550900405|ref|YP_008669497.1	phage lysozyme	C
gi|550900417|ref|YP_008669509.1	tape measure protein	C
gi|550900428|ref|YP_008669520.1	Tail Fiber Protein	A
gi|550900558|ref|YP_008669650.1	flagellar protein FlgJ	C

gi|550900559|ref|YP_008669651.1	flagellar hook-associated protein 1	C
gi|550900581|ref|YP_008669673.1	outer-membrane receptor	C
gi|550900655|ref|YP_008669747.1	outer membrane protein	C
gi|550900947|ref|YP_008670039.1	TonB-dependent receptor YncD	A
gi|550900982|ref|YP_008670074.1	glucans biosynthesis protein D	C
gi|550901038|ref|YP_008670130.1	periplasmic murein peptide-binding protein MppA	C
gi|550901039|ref|YP_008670131.1	hypothetical protein DT104_16501	A
gi|550901155|ref|YP_008670247.1	hypothetical protein DT104_17681	B
gi|550901233|ref|YP_008670325.1	prophage protein	A
gi|550901239|ref|YP_008670331.1	transglycosylase	C
gi|550901253|ref|YP_008670345.1	Lysozome	C
gi|550901388|ref|YP_008670480.1	outer membrane protein S1	A
gi|550901606|ref|YP_008670698.1	penicillin-binding protein	B
gi|550901817|ref|YP_008670909.1	penicillin-insensitive murein endopeptidase	B
gi|550901877|ref|YP_008670969.1	N-acetylmuramoyl-L-alanine amidase	A
gi|550901905|ref|YP_008670997.1	hypothetical protein DT104_25311	A
gi|550901939|ref|YP_008671031.1	host colonisation factor ShdA	A
gi|550901940|ref|YP_008671032.1	protein RatA	A
gi|550901990|ref|YP_008671082.1	hypothetical protein DT104_26181	C
gi|550902014|ref|YP_008671106.1	DNA recombinase	A
gi|550902021|ref|YP_008671113.1	prophage protein	C
gi|550902038|ref|YP_008671130.1	prophage protein	C
gi|550902039|ref|YP_008671131.1	phage encoded lysozyme	C
gi|550902109|ref|YP_008671201.1	large repetitive protein	A
gi|550902196|ref|YP_008671288.1	membrane-bound lytic murein transglycosylase B	C
gi|550902241|ref|YP_008671333.1	cell invasion protein	D
gi|550902354|ref|YP_008671446.1	N-acetylmuramoyl-L-alanine amidase	A
gi|550902474|ref|YP_008671566.1	membrane-bound lytic murein transglycosylase C	C
gi|550902482|ref|YP_008671574.1	arylsulfatase	C

gi|550902655|ref|YP_008671747.1	penicillin-binding protein	B
gi|550902823|ref|YP_008671915.1	surface-exposed virulence protein BigA	A
gi|550902985|ref|YP_008672077.1	lipoprotein	A
gi|550902988|ref|YP_008672080.1	biotin sulfoxide reductase	A
gi|550903005|ref|YP_008672097.1	amidase	C
gi|550903100|ref|YP_008672192.1	autotransported protein MisL	A
gi|550903489|ref|YP_008672581.1	vitamin B12 receptor protein	A
gi|550903561|ref|YP_008672653.1	hypothetical protein DT104_42111	C
gi|550903695|ref|YP_008672787.1	N-acetylmuramoyl-L-alanine amidase	A
gi|550903909|ref|YP_008673001.1	lytic murein transglycosylase	C

Shigella flexneri 2a str. 2457T		
**GI ID**	**Protein**	***Class**
gi|30061676|ref|NP_835847.1	N-acetyl-anhydromuranmyl-L-alanine amidase	A
gi|30061763|ref|NP_835934.1	membrane-bound lytic murein transglycosylase D	C
gi|30061781|ref|NP_835952.1	endolysin R of prophage CP-933V, partial	C
gi|30061800|ref|NP_835971.1	DNA transfer protein	C
gi|30061837|ref|NP_836008.1	Rhs-family protein	C
gi|30062230|ref|NP_836401.1	endolysin R of prophage CP-933V	C
gi|30062231|ref|NP_836402.1	endopeptidase	B
gi|30062298|ref|NP_836469.1	outer membrane protein X	C
gi|30062351|ref|NP_836522.1	regulator	A
gi|30062460|ref|NP_836631.1	hypothetical protein S0986	C
gi|30062617|ref|NP_836788.1	flagellar rod assembly protein/muramidase FlgJ	C
gi|30062687|ref|NP_836858.1	hypothetical protein S1242	A
gi|30062710|ref|NP_836881.1	L,D-carboxypeptidase A	B
gi|30062726|ref|NP_836897.1	adhesion and penetration protein	A
gi|30062815|ref|NP_836986.1	peptide transport periplasmic protein	C
gi|30062844|ref|NP_837015.1	murein peptide amidase A	A

gi|30062850|ref|NP_837021.1	transport periplasmic protein	C
gi|30063011|ref|NP_837182.1	hypothetical protein S1625	C
gi|30063541|ref|NP_837712.1	hypothetical protein S2289	C
gi|161486468|ref|NP_837752.2	D-alanyl-D-alanine endopeptidase	B
gi|30063753|ref|NP_837924.1	hypothetical protein S2538	B
gi|30063754|ref|NP_837925.1	penicillin-insensitive murein endopeptidase	B
gi|30063765|ref|NP_837936.1	long-chain fatty acid outer membrane transporter	C
gi|30063827|ref|NP_837998.1	N-acetylmuramoyl-l-alanine amidase I	A
gi|30063868|ref|NP_838039.1	hypothetical protein S2687	C
gi|30063945|ref|NP_838116.1	transglycosylase	C
gi|30063958|ref|NP_838129.1	hypothetical protein S2790	C
gi|30064031|ref|NP_838202.1	LysM domain/BON superfamily protein	C
gi|30064061|ref|NP_838232.1	murein hydrolase B	C
gi|30064159|ref|NP_838330.1	amidase	A
gi|30064284|ref|NP_838455.1	murein transglycosylase C	C
gi|30064293|ref|NP_838464.1	serine protease precurser	A
gi|30064520|ref|NP_838691.1	D-alanyl-D-alanine carboxypeptidase	B
gi|30064743|ref|NP_838914.1	vitamin B12/cobalamin outer membrane transporter	A
gi|30065145|ref|NP_839316.1	hypothetical protein S4155	C
gi|30065417|ref|NP_839588.1	hypothetical protein S4448	C
gi|30065527|ref|NP_839698.1	beta-lactamase	B
gi|30065541|ref|NP_839712.1	N-acetylmuramoyl-l-alanine amidase II	A
gi|161486362|ref|NP_839800.2	lytic murein transglycosylase	C

Staphylococcus aureus subsp. aureus ST228		
**GI ID**	**Protein**	***Class**
gi|470221981|ref|YP_007620538.1	Lysostaphin	A
gi|470222107|ref|YP_007620664.1	N-acetylmuramoyl-L-alanine amidase sle1	A
gi|470222266|ref|YP_007620823.1	Secretory antigen SsaA homologue	A

gi|470222491|ref|YP_007621048.1	Bifunctional autolysin	C
gi|470222773|ref|YP_007621330.1	C-terminal processing peptidase family protein	A
gi|470222931|ref|YP_007621488.1	Probable cell wall amidase LytH	A
gi|470223049|ref|YP_007621606.1	Family=4 N-acetylmuramoyl-L-alanine amidase	C
gi|470223179|ref|YP_007621736.1	Lytic enzyme	A
gi|470223183|ref|YP_007621740.1	Bacteriophage tail length tape measure protein	D
gi|470223386|ref|YP_007621943.1	Urease subunit alpha	A
gi|470223393|ref|YP_007621950.1	Staphylococcal secretory antigen ssaA2	A
gi|470223607|ref|YP_007622164.1	Staphylococcal secretory antigen ssaA	A
gi|470223610|ref|YP_007622167.1	Probable transglycosylase isaA	C
gi|470223677|ref|YP_007622234.1	Similar to autolysin	C

Staphylococcus epidermidis ATCC 12228		
**GI ID**	**Protein**	***Class**
gi|27467163|ref|NP_763800.1	triacylglycerol lipase	A
gi|27467351|ref|NP_763988.1	secretory antigen SsaA-like protein	A
gi|27467455|ref|NP_764092.1	hypothetical protein SE0537	A
gi|27467668|ref|NP_764305.1	N-acetylmuramoyl-L-alanine amidase	A
gi|27468231|ref|NP_764868.1	N-acetylmuramoyl-L-alanine amidase	A
gi|27468361|ref|NP_764998.1	hypothetical protein SE1443	A
gi|27468790|ref|NP_765427.1	ssaA protein	A
gi|27469042|ref|NP_765679.1	ssaA protein	A
gi|27469044|ref|NP_765681.1	hypothetical protein SE2126	C
gi|27469115|ref|NP_765752.1	alkaline phosphatase	A
gi|27469149|ref|NP_765786.1	N-acetylmuramoyl-L-alanine amidase	A
gi|27469237|ref|NP_765874.1	autolysin (N-acetylmuramoyl-L-alanine amidase	A
gi|27469291|ref|NP_765928.1	1,4-beta-N-acetylmuramidase	C

Streptococcus pneumoniae ATCC 700669		

GI ID	Protein	*Class
gi|221231241|ref|YP_002510393.1	cell wall surface anchored protein	A
gi|221231242|ref|YP_002510394.1	penicillin-binding protein 1A	C
gi|221231747|ref|YP_002510899.1	glycosyl hydrolase	A
gi|221232001|ref|YP_002511153.1	sialidase (neuraminidase)	A
gi|221232082|ref|YP_002511235.1	cell wall hydrolase	B
gi|221232275|ref|YP_002511428.1	autolysin	A
gi|221232276|ref|YP_002511429.1	antiholin	A
gi|221232280|ref|YP_002511433.1	platelet-binding phage protein	A
gi|221232334|ref|YP_002511487.1	exported choline-binding glycosyl hydrolase	C
gi|221232666|ref|YP_002511820.1	cell wall-binding amidase	A

Streptococcus pyogenes A20		
**GI ID**	**Protein**	***Class**
gi|410679721|ref|YP_006932123.1	hypothetical protein A20_0061c	A
gi|410680179|ref|YP_006932581.1	glycosyl hydrolases 25 family protein	A
gi|410680343|ref|YP_006932745.1	mannosyl-glycoendo-beta-N-acetylglucosaminidase family protein	C
gi|410680669|ref|YP_006933071.1	phage holin, LL-H family	A
gi|410680674|ref|YP_006933076.1	mannosyl-glycoendo-beta-N-acetylglucosaminidase family protein	A
gi|410680732|ref|YP_006933134.1	cyclomaltodextrin glucanotransferase	A
gi|410680839|ref|YP_006933241.1	mannosyl-glycoendo-beta-N-acetylglucosaminidase family protein	A
gi|410681018|ref|YP_006933420.1	penicillin-binding protein 1A	C
gi|410681200|ref|YP_006933602.1	CHAP domain-containing protein	A
gi|410681339|ref|YP_006933741.1	pullulanase	A
gi|410681372|ref|YP_006933774.1	C5a peptidase	A
gi|410681408|ref|YP_006933810.1	penicillin-binding , 1A family protein	C
gi|410681497|ref|YP_006933899.1	transglycosylase-like domain-containing protein	C

Treponema pallidum subsp. pallidum str. Nichols		

GI ID	Protein	*Class
gi|15639037|ref|NP_218483.1	soluble lytic transglycosylase,	C
gi|15639239|ref|NP_218687.1	N-acetylmuramoyl-L-alanine amidase (amiA)	A
gi|15639560|ref|NP_219010.1	Tp70 protein	C
gi|15639793|ref|NP_219243.1	femA protein,	C

Vibrio cholerae O395		
**GI ID**	**Protein**	***Class**
gi|147673807|ref|YP_001216126.1	D-alanyl-D-alanine carboxypeptidase	B
gi|147673203|ref|YP_001216193.1	soluble lytic murein transglycosylase	C
gi|147674148|ref|YP_001216207.1	protease	B
gi|147673383|ref|YP_001216326.1	lipoprotein	A
gi|147674227|ref|YP_001216347.1	transglycosylase	C
gi|147674390|ref|YP_001216595.1	hypothetical protein VC0395_A0645	C
gi|147673728|ref|YP_001216638.1	bacteriophage Mu tail sheath protein (GpL)	C
gi|147674078|ref|YP_001216699.1	bacteriophage Mu tail sheath protein (GpL)	C
gi|147675379|ref|YP_001217171.1	cadherin domain-containing protein	A
gi|147673281|ref|YP_001217324.1	neuraminidase	A
gi|147675561|ref|YP_001217481.1	chitinase	C
gi|147673804|ref|YP_001217753.1	membrane-bound lytic murein transglycosylase D	C
gi|147673296|ref|YP_001217921.1	N-acetyl-anhydromuranmyl-L-alanine amidase	A
gi|147674843|ref|YP_001218242.1	TonB-dependent receptor	C
gi|147675682|ref|YP_001218613.1	N-acetylmuramoyl-L-alanine amidase	A

Yersinia pestis A1122		
**GI ID**	**Protein**	***Class**
gi|384137037|ref|YP_005519739.1	putative kinase	B
gi|384137161|ref|YP_005519863.1	lac repressor	A
gi|384137347|ref|YP_005520049.1	outer membrane channel protein	C

gi|384137406|ref|YP_005520108.1	putative autotransporter protein	C
gi|384137413|ref|YP_005520115.1	putative adhesin	C
gi|384137424|ref|YP_005520126.1	putative autotransporter protein	A
gi|384137509|ref|YP_005520211.1	hypothetical protein A1122_02570	C
gi|384137517|ref|YP_005520219.1	LPS assembly outer membrane complex protein LptD	A
gi|384137559|ref|YP_005520261.1	lytic murein transglycosylase	C
gi|384137648|ref|YP_005520350.1	N-acetylmuramoyl-L-alanine amidase-family protein	A
gi|384137711|ref|YP_005520413.1	hypothetical protein A1122_03590	A
gi|384138154|ref|YP_005520856.1	periplasmic pectate lyase	C
gi|384138224|ref|YP_005520926.1	putative TonB dependent receptor protein	A
gi|384138237|ref|YP_005520939.1	vitamin B12/cobalamin outer membrane transporter	C
gi|384138442|ref|YP_005521144.1	hypothetical protein A1122_07345	C
gi|384138470|ref|YP_005521172.1	Rhs-like core protein	A
gi|384138643|ref|YP_005521345.1	D-alanyl-D-alanine carboxypeptidase	B
gi|384138726|ref|YP_005521428.1	N-acetyl-anhydromuranmyl-L-alanine amidase	A
gi|384138915|ref|YP_005521617.1	putative adhesin	A
gi|384139126|ref|YP_005521828.1	putative autotransporter	A
gi|384139196|ref|YP_005521898.1	murein hydrolase B	C
gi|384139337|ref|YP_005522039.1	membrane-bound lytic transglycosylase F	C
gi|384139363|ref|YP_005522065.1	putative autotransporter protein	A
gi|384139407|ref|YP_005522109.1	penicillin-insensitive murein endopeptidase	B
gi|384139431|ref|YP_005522133.1	hypothetical protein A1122_12405	C
gi|384139508|ref|YP_005522210.1	putative solute-binding protein	A
gi|384139522|ref|YP_005522224.1	glycine betaine transporter periplasmic subunit	C
gi|384139668|ref|YP_005522370.1	outer membrane protein X	C
gi|384139684|ref|YP_005522386.1	putative hemolysin	A
gi|384139737|ref|YP_005522439.1	putative membrane-bound lytic murein transglycosylase	C
gi|384139793|ref|YP_005522495.1	insecticidal toxin	A
gi|384139833|ref|YP_005522535.1	murein peptide amidase A	B

gi|384139965|ref|YP_005522667.1	bifunctional indole-3-glycerol phosphate synthase/phosphoribosylanthranilate isomerase	C
gi|384140017|ref|YP_005522719.1	hypothetical protein A1122_15380	C
gi|384140037|ref|YP_005522739.1	putative phage host specificity protein	A
gi|384140072|ref|YP_005522774.1	putative phage lysozyme	C
gi|384140126|ref|YP_005522828.1	putative hemolysin	A
gi|384140230|ref|YP_005522932.1	hypothetical protein A1122_16460	C
gi|384140302|ref|YP_005523004.1	flagellar hook-associated protein FlgK	C
gi|384140303|ref|YP_005523005.1	flagellar rod assembly protein/muramidase FlgJ	C
gi|384140398|ref|YP_005523100.1	N-acetylmuramoyl-L-alanine amidase	A
gi|384140431|ref|YP_005523133.1	N-acetylmuramoyl-L-alanine amidase	A
gi|384140439|ref|YP_005523141.1	hypothetical protein A1122_17520	A
gi|384140886|ref|YP_005523588.1	D-alanyl-D-alanine endopeptidase	B
gi|384140913|ref|YP_005523615.1	outer membrane protein C2	C
gi|384140939|ref|YP_005523641.1	outer membrane protease	C
gi|384140950|ref|YP_005523652.1	putative bacteriophage tail sheath protein	A
gi|384141012|ref|YP_005523714.1	outer membrane usher protein PsaC	A
gi|384141110|ref|YP_005523812.1	N-acetylmuramoyl-L-alanine amidase	A
gi|384141122|ref|YP_005523824.1	putative TonB-dependent outer membrane receptor	C
